# Curcumol Ameliorates Diabetic Nephropathy by Inhibiting Podocyte Ferroptosis Through the xCT/GPX4 Pathway

**DOI:** 10.1155/jdr/5577736

**Published:** 2026-03-31

**Authors:** Yue Ji, Yuqi Wu, Jingyi Tang, Yachun Li, Xi Guo, Jiakun Zhang, Weijun Huang, Yuning Liu, Zhongjie Liu, Wei Jing Liu

**Affiliations:** ^1^ Institute of Nephrology & Beijing Key Laboratory, Dongzhimen Hospital, Beijing University of Traditional Chinese Medicine, Beijing, China, bucm.edu.cn; ^2^ Nephrology Department, Shaanxi Provincial Hospital of Chinese Medicine, Xi′an, China

**Keywords:** curcumol, diabetic kidney disease, ferroptosis, xCT/GPX4 pathway

## Abstract

**Background:**

Diabetic nephropathy is a leading complication of diabetes mellitus and poses a significant public health challenge. Ferroptosis has emerged as a critical pathological factor that exacerbates the progression of diabetic nephropathy. While previous studies have demonstrated the antiferroptotic effects of curcumol (Cur), its therapeutic potential in treating diabetic nephropathy, along with the underlying mechanisms, remains to be fully elucidated.

**Methods:**

To investigate this, we established a high glucose‐induced MPC‐5 cell injury model. Initially, we identified the safe concentration range of curcumol. Using Western blot (WB) analysis and transcriptomic profiling, we assessed target proteins and regulatory pathways, and systematically measured ferroptosis‐related biomarkers. To further explore Cur′s effect on ferroptosis, we cotreated the in vitro model with the ferroptosis inhibitor Fer‐1 and activator RLS3, analyzing the key pathways involved using WB and transcriptomic approaches. Additionally, we established a diabetic kidney disease (DKD) mouse model to assess the effects of Cur on renal function indicators, including serum creatinine, urea nitrogen, and 24‐h urinary protein levels. Renal pathological changes and molecular markers were evaluated, and the core pathway mechanisms were validated by WB analysis.

**Results:**

In in vitro experiments, Cur reduced iron deposition and total iron content by downregulating TRF and NCOA4. It also inhibited ACSL4‐mediated lipid peroxidation, resulting in lower levels of ROS, MDA, and 4‐HNE. Additionally, Cur upregulated the expression of SLC3A2, SLC7A11, and GPX4, thereby restoring the GSH‐GPX4 antioxidant system. In vivo, Cur improved renal function and alleviated renal injury in DKD mice through the xCT/GPX4 signaling pathway. These findings suggest that Cur mitigates ferroptosis via the xCT/GPX4 pathway, ultimately slowing the progression of diabetic kidney disease.

**Conclusion:**

Our in vitro and in vivo experiments demonstrate that Cur has therapeutic potential for DKD. Mechanistically, Cur protects against podocyte ferroptosis by modulating the xCT/GPX4 pathway.

## 1. Introduction

Diabetic kidney disease (DKD), a chronic renal condition triggered by diabetes mellitus, represents one of the most severe microvascular complications associated with diabetes. It involves complex pathogenic mechanisms, persistent elevation in albuminuria, and/or a progressive decline in glomerular filtration rate (GFR), which may ultimately lead to end‐stage renal disease (ESRD) [1]. A nationwide cross‐sectional study in China reported that among adult diabetic patients, the prevalence rates of chronic kidney disease, albuminuria, and reduced estimated GFR (eGFR) were 32.6%, 30.8%, and 5.5%, respectively [2]. Current therapeutic approaches for managing DKD, in addition to symptomatic treatments aimed at regulating glucose and lipid metabolism, include the use of sodium–glucose cotransporter 2 (SGLT2) inhibitors, glucagon‐like peptide‐1 (GLP‐1) receptor agonists, and mineralocorticoid receptor antagonists (MRAs). However, each of these strategies has notable limitations. Consequently, there is a critical need to explore the potential of traditional Chinese medicine, develop novel therapeutic agents to improve DKD, and provide alternative strategies for its treatment.

Podocytes are terminally differentiated, highly specialized visceral epithelial cells within the glomerulus [3]. Ferroptosis is an iron‐dependent form of regulated cell death, characterized by disturbances in iron metabolism, lipid peroxidation, and an imbalance in the antioxidant system [4]. GPX4 (glutathione peroxidase 4) serves as a key intracellular regulator that maintains redox homeostasis and is thus critically involved in ferroptosis [5]. System Xc^-^ is a disulfide‐linked heterodimer composed of a light chain subunit (SLC7A11, xCT) and a heavy chain subunit (SLC3A2). Glutathione (GSH), an intracellular antioxidant, participates in both enzymatic and nonenzymatic reactions to maintain physiological levels of hydrogen peroxide in cells. The xCT subunit, located on the plasma membrane, facilitates the uptake of cystine, which provides a crucial raw material for the synthesis of GSH. GSH, in turn, acts as an essential cofactor for GPX4, enabling it to eliminate lipid peroxides and thereby execute its central function in suppressing ferroptosis [4]. It is an important cause of podocyte injury [6].

Studies have shown that compared with healthy individuals, patients with DKD exhibit higher levels of systemic iron overload [7] along with increased intracellular accumulation of reactive oxygen species (ROS) and lipid peroxides [8]. This labile iron contributes to excessive ROS generation, thereby triggering autophagy and apoptosis, and ultimately leading to ferroptosis [9]. The progression of DKD may be linked to the occurrence of ferroptosis. Furthermore, high‐fructose conditions can exacerbate podocyte damage, and alleviating ferroptosis [10] has been shown to effectively reduce podocyte injury in DKD [11].

Extensive research has explored the role of individual Chinese medicine monomers in combating podocyte ferroptosis. For example, astragalus polysaccharides promote M2 macrophage polarization, reduce pro‐inflammatory cytokines, and inhibit ferroptosis via the Nrf2/HO‐1/SLC7A11 axis [12]. Investigating the mechanisms by which Chinese medicine monomers influence podocyte ferroptosis in the context of DKD may provide novel insights for DKD treatment strategies.

Curcumol, derived from the traditional Chinese herb E Zhu (Rhizome Curcumae), has garnered attention for its therapeutic potential. Studies indicate that curcumol, in combination with icariin, induces ferroptosis in prostate cancer (PCa) cells by inhibiting the Nrf2/HO‐1 signaling pathway [13]. Additionally, curcumol has been shown to activate endoplasmic reticulum stress, correct disturbances in glucose and lipid metabolism, and exert beneficial effects in the context of obesity [14]. Moreover, curcumol promotes autophagy in hepatic stellate cells, facilitates the degradation of NCOA4‐FTH1 complexes, and triggers the release of total iron. This process leads to iron overload, induces ferroptosis, and contributes to its antihepatic fibrosis effects [15]. Given that diabetic nephropathy shares common pathological features, including metabolic disturbances in glucose and lipids and renal fibrosis, it is critical to explore whether curcumol can exert antiferroptotic effects in the treatment of DKD.

In this study, we first established an in vitro high–glucose‐induced MPC‐5 cell injury model to evaluate the therapeutic effects of curcumol. Transcriptomic analysis was then employed to investigate curcumol‐induced changes in gene expression in high–glucose‐treated MPC‐5 cells, aiming to identify potential molecular targets. Validation experiments were conducted to elucidate the underlying molecular mechanisms by which curcumol inhibits ferroptosis. Next, we developed a mouse model of DKD to assess renal function, pathological damage, and indicators of renal iron deposition. This in vivo approach allowed us to evaluate curcumol′s efficacy in mitigating DKD and its capacity to counteract ferroptosis. This research is aimed at providing experimental evidence supporting the development of multitarget therapeutic strategies for DKD and to contribute to the clinical application of curcumol.

## 2. Materials and Methods

### 2.1. Cells, Animals, and Reagents

MPC‐5 cells were sourced from Wuhan Pricella Biotechnology Co. Ltd. (Catalog No.CL‐0855). Healthy male C57BL/6 mice, aged 6–7 weeks, were obtained from SiPeiFu (production license No.: SYXK [Beijing] 2019‐0030). All animal experiments received approval from the Ethics Committee of Dongzhimen Hospital, Beijing University of Chinese Medicine (Approval No.: DZMYY‐24‐07). Details of the kits and antibodies used in the experiments are provided in the supporting information.

### 2.2. In Vitro Experiments

#### 2.2.1. Cell Culture, Model Establishment, Selection of Activators/Inhibitors, and Grouping

MPC‐5 cells were cultured in DMEM supplemented with 10% fetal bovine serum (FBS) and 1% penicillin‐streptomycin (P/S) and maintained in a 37°C incubator with 5% CO_2_ under saturated humidity. The culture medium was refreshed 2–3 times per week, and passaging was performed at a ratio of 1:2–1:4. To establish the high–glucose‐induced MPC‐5 injury model, cells were treated with glucose concentrations of 0, 50, 100, 200, 400, and 800 mM for 24 h. The optimal glucose concentration for model induction was determined using the MTT assay.

To determine the nontoxic dose of Cur for MPC‐5 cells, we exposed the cells to Cur concentrations of 0, 6.25, 12.5, 25, 50, and 100 *μ*M for 24 h. The nontoxic dose was identified using the MTT assay. To assess Cur′s effect on high–glucose‐induced MPC‐5 cell injury, we divided the cells into the following groups: control, control + H − Cur, HG (400 mM glucose), HG + L − Cur (12.5 *μ*M), HG + M − Cur (25 *μ*M), and HG + H − Cur (50 *μ*M), with each group treated for 24 h. Cells from the control, HG, and HG + H − Cur groups were then selected for transcriptomic analysis, and ferroptosis‐related indicators were measured.

To validate the antiferroptotic effect of Cur, we treated MPC‐5 cells with the ferroptosis activator RSL3 (0.5 *μ*mol/L) [16, 17] and the ferroptosis inhibitor Fer‐1 (20 *μ*mol/L) [18] in combination with high‐glucose exposure and high‐dose Cur (50 *μ*M) intervention. After 6 h of treatment, we assessed ferroptosis‐related markers. The cells were divided into the following groups: control, HG, HG + H − Cur, HG + Fer − 1, HG + RSL3, and HG + RSL3 + Cur (50 *μ*M).

#### 2.2.2. MTT

After seeding the MPC‐5 cells, we performed the interventions according to the protocol outlined in Section 2.2.1 The cells were incubated at 37°C with 5% CO_2_ for 24 h. Following incubation, 10 *μ*L of MTT solution (5 mg/mL) was added to each well. After an additional 4‐h incubation, the supernatant was discarded, and 150 *μ*L of DMSO was added to each well. The crystals were dissolved by gently oscillating the plates, and absorbance was measured at 490 nm using a microplate reader.

#### 2.2.3. Transcriptomics

Transcriptomics was performed by Tianjin Genink biotechnology Co.,Ltd. Cells from each group were collected, and total RNA was extracted. The RNA quality was rigorously assessed through purity evaluation, concentration quantification, and integrity verification. Samples that passed quality control were used for library construction, followed by deep transcriptome sequencing on the Illumina platform. For bioinformatics analysis, we applied the DESeq2 algorithm. Differentially expressed genes (DEGs) were identified by comparing the HG and control groups, as well as the H‐Cur and HG groups. The selection criteria for DEGs were set as: |Log2(Fold Change)| ≥ 1 and adjusted *p* value (p_adj_) ≤ 0.05. Pathway enrichment analysis of the identified DEGs was then performed using the KEGG database.

### 2.3. In Vivo Experiments

#### 2.3.1. Model Establishment, Grouping, and Administration

We randomly selected 10 mice from a total of 60 as the control group, with the remaining 50 mice used to establish the DKD mouse model [19]. These mice were first fed a high‐fat diet (HFD) for eight weeks, followed by an intraperitoneal injection of STZ at a dose of 30 mg/kg. Three days later, we measured the fasting blood sugar levels of the mice. Type 2 diabetes mellitus (T2DM) was confirmed when blood glucose levels reached ≥ 16.7 mmol/L. After continued feeding, we measured 24‐h urinary total protein (24 h‐UTP) of mice once a week. The criteria for DKD modeling were defined as a blood glucose level ≥ 16.7 mmol/L and 24h urinary total protein (24 h‐UTP) ≥ 20 mg. Upon successful model establishment, the 50 mice were randomly divided into five groups: the model group (DKD), irbesartan (IRB) group, low‐dose Cur group (L‐Cur), medium‐dose Cur group (M‐Cur), and high‐dose Cur group (H‐Cur), with 10 mice per group. The control and DKD groups received 0.2 mL of normal saline daily via gavage. The IRB group was administered IRB (0.2 g/kg per day) via gavage, whereas the L‐Cur, M‐Cur, and H‐Cur groups were treated with Cur at doses of 15, 30, and 60 mg·kg^−1^·d^−1^, respectively [20].

We administered all treatments continuously for 8 weeks. During this period, we measured and recorded the body weight and random blood glucose levels of each mouse weekly. After 8 weeks, all mice were fasted for 12 h. We collected 24‐h urine samples in metabolic cages, centrifuged the samples to obtain the supernatant, and stored them at −80°C.

Following anesthesia with 5% Isoflurane (RWD R510‐22‐10) using an inhalation anesthesia system, we performed retro‐orbital bleeding by gently protruding the eye and using a capillary tube to collect blood from the retro‐orbital sinus. The blood samples were centrifuged to obtain the supernatant and stored at −80°C. Subsequently, the animals were euthanized by cervical dislocation. We fixed the left kidney in 4% paraformaldehyde for histopathological examination and snap‐froze the right kidney in liquid nitrogen for storage at −80°C pending further experimental analysis.

#### 2.3.2. Detection of Renal Function Indicators

Urine samples from all groups of mice were collected, and the 24 h‐UTP level was measured following the manufacturer′s instructions. Mice were anesthetized with sodium Isoflurane. Following weighing, blood samples of 0.5–0.8 ml were collected from the eyeball. Blood samples were then centrifuged at 400 g for 15 min to obtain serum. Serum levels of creatinine (Cr) and blood urea nitrogen (BUN) were measured according to the specifications provided in the respective kits.

#### 2.3.3. Pathological Staining

Pathological Staining was performed by Beijing Jinke perserbio Co.,Ltd and Tianjin Genink biotechnology Co.,Ltd. Collect kidney tissue from mice and remove blood and impurities using physiological saline. The left kidneys from mice in each group were harvested, fixed, dehydrated, and embedded in paraffin. Kidney sections of 4 *μ*m thickness were then prepared. The sections underwent staining with hematoxylin‐eosin (HE), periodic acid‐Schiff (PAS), and Prussian blue (BP) as previously described. The stained sections were observed under a light microscope, and the results were analyzed through scoring or quantitative methods [21].

### 2.4. Detection of Lipid Oxidation

For in vivo experiments, renal tissues were collected from each group of mice, homogenized, and the total protein content was determined using the BCA assay. For in vitro experiments, the levels of ROS, malondialdehyde (MDA), 4‐hydroxynonenal (4‐HNE), and the GSH/glutathione disulfide (GSSG) ratio were measured using appropriate kits.

### 2.5. Detection of Total Iron Content

Renal tissues and cells from each group were collected and homogenized. After determining the total protein concentration using the BCA assay, total iron content was quantified following the kit instructions.

### 2.6. Western blot

Total proteins were extracted from renal tissues or cells, and their concentrations were determined using the BCA assay. Following SDS‐PAGE separation on precast gels (LABLEAD, P41215, P00815), proteins were transferred to membranes and blocked. Primary antibodies targeting the proteins of interest were added, and the membranes were incubated overnight at 4°C. Following this, HRP‐conjugated secondary antibodies were applied and incubated at room temperature for 2 h. ECL reagents were then added, and protein bands were visualized using an automatic gel imaging system. The expressions of SLC3A2, SLC7A11, GPX4, TRF, NCOA4, and ACSL4 proteins have been detected. *β*‐ACTIN was used as the internal reference, and the gray values of the protein bands were quantified and analyzed using Image J.

### 2.7. Statistical Analysis

Statistical analyses were performed using SPSS 25.0 software. Data are presented as mean ± standard deviation (SD). To compare two groups, we used Student′s *t*‐test; for multiple group comparisons, one‐way analysis of variance (ANOVA) was employed, followed by Tukey′s post hoc test. A *p* value < 0.05 was considered statistically significant.

## 3. Results

### 3.1. Cur Intervention Alleviates High–Glucose‐Induced MPC‐5 Cell Injury

In the in vitro experiments, we first exposed MPC‐5 cells to varying glucose concentrations to identify the optimal level for inducing high–glucose‐mediated injury. As shown in Figure 1a, in comparison with 0 mM glucose, the MTT assay revealed that treating cells with 400 mM glucose reduced cell viability to approximately 60% (*p* < 0.01), whereas 800 mM glucose decreased viability to about 30% (*p* < 0.01). Glucose at concentrations of up to 200 mM showed no significant effect on cell viability (*p* > 0.05). Based on these results, 400 mM glucose was selected for subsequent experiments to induce MPC‐5 cell injury.

Figure 1Cur intervention protects MPC‐5 cells against high–glucose‐induced injury. (a) To determine the optimal concentration for establishing high–glucose‐induced MPC‐5 injury models, glucose at concentrations of 0, 50, 100, 200, 400, and 800 mM was used to treat MPC‐5 cells for 24 h. MTT assay results showed that 400 mM glucose reduced MPC‐5 cell viability by approximately 40%, making it the most suitable for inducing MPC‐5 cell injury under high‐glucose conditions. (b) To screen for nontoxic doses, Cur at concentrations of 0, 6.25, 12.5, 25, 50, and 100 *μ*M was applied to MPC‐5 cells for 24 h. MTT results indicated that Cur at concentrations below 50 *μ*M did not significantly reduce MPC‐5 cell viability; thus, 12.5, 25, and 50 *μ*M Cur were selected for subsequent experiments. (c) Cur intervention increases the viability of high–glucose‐induced MPC‐5 cells in a dose‐dependent manner. (d–g) Cur intervention decreases levels of ROS (d), MDA (e), and 4‐HNE (f) in high–glucose‐induced MPC‐5 cells, and upregulates the GSH/GSSG ratio (g). Data are presented as means ± SD. *n* = 6 biologically independent samples for a–g. When data exhibit a normal distribution with homogeneity of variance, we use one‐way analysis of variance (ANOVA). When data exhibit a nonnormal distribution with heterogeneity of variance, we use the Kruskal–Wallis (KW) test. ∗*p* < 0.05, ∗∗*p* < 0.01. #*p* < 0.05, ##*p* > 0.05.

**Figure:**
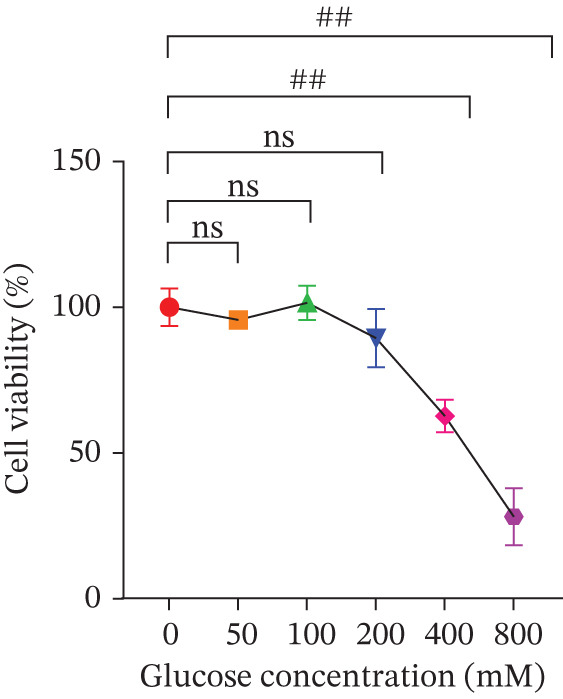
(a)

**Figure:**
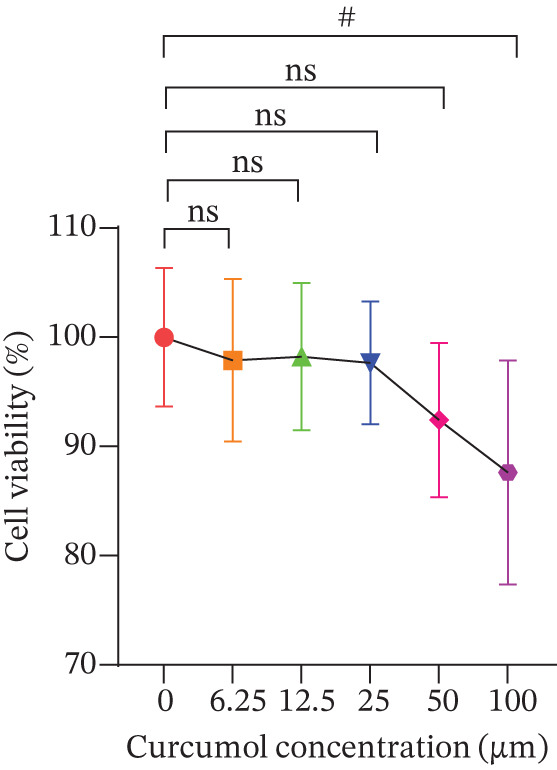
(b)

**Figure:**
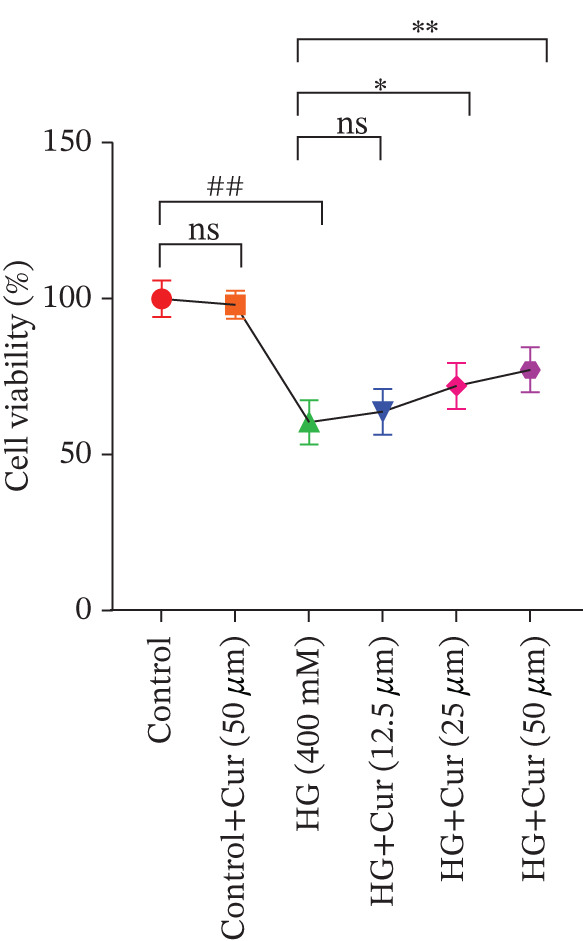
(c)

**Figure:**
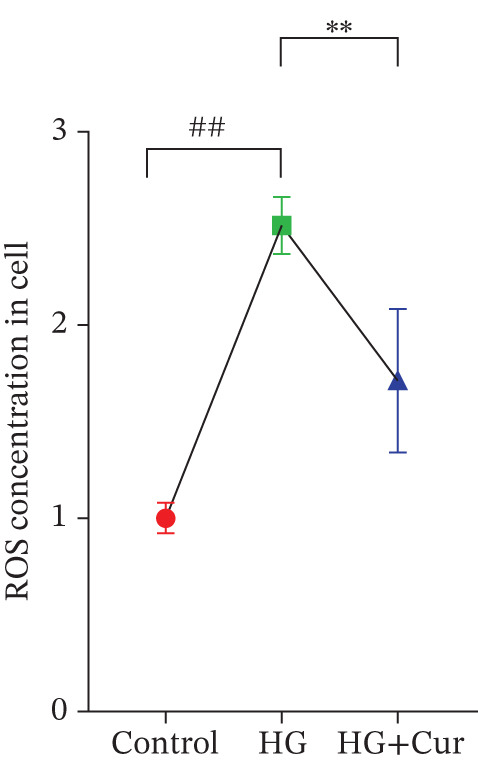
(d)

**Figure:**
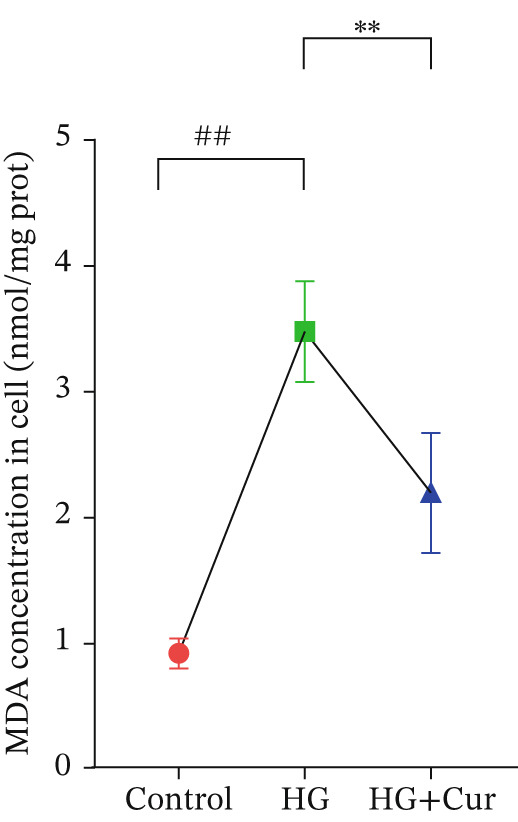
(e)

**Figure:**
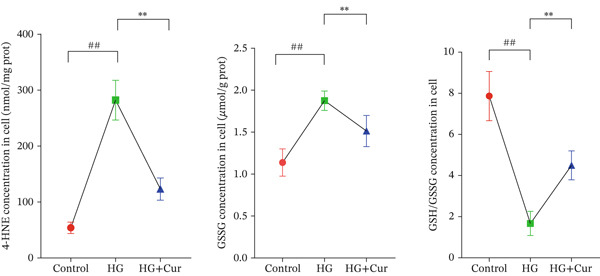
(f)

**Figure:**
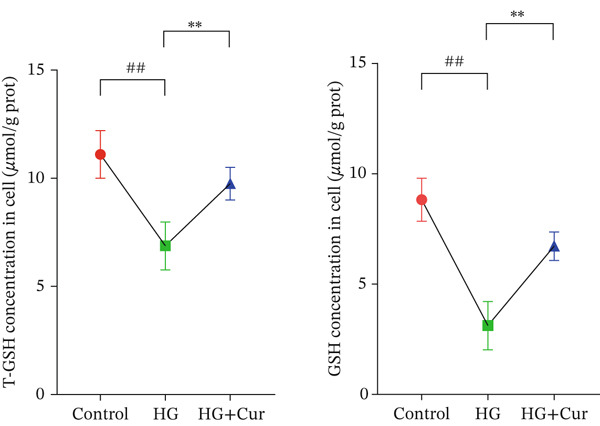
(g)

Next, we treated MPC‐5 cells with different concentrations of Cur to determine noncytotoxic doses. As depicted in Figure 1b, the MTT assay indicated that Cur concentrations up to 50 *μ*M did not significantly reduce cell viability (*p* > 0.05), whereas 100 *μ*M Cur led to a marked decrease in viability (*p* < 0.05). Therefore, we selected 12.5, 25, and 50 *μ*M Cur for further evaluation of its therapeutic effects on high–glucose‐induced MPC‐5 cell injury.

Figure 1c demonstrates that high‐glucose treatment significantly reduced MPC‐5 cell viability (*p* < 0.01), whereas Cur treatment restored cell viability in a dose‐dependent manner. High‐glucose intervention significantly increased the levels of ROS, MDA, and 4‐HNE (*p* < 0.01) and decreased the GSH/GSSG ratio (*p* < 0.01).

The analysis of cell (Figures 1d, 1e, 1f, and 1g) revealed that H‐Cur intervention lowered the levels of ROS, MDA, and 4‐HNE in the high–glucose‐treated cells (*p* < 0.01), whereas it increased the GSH/GSSG ratio (*p* < 0.01).

### 3.2. Effects of Cur Intervention on Transcriptomics of High–Glucose‐Induced MPC‐5 Cells

Transcriptomic analysis (Figure 2a,b) was performed on cells from the control, HG, and HG + H − Cur groups (schematic diagram). DEGs were identified for HG vs. control and HG + Cur vs. HG using the criteria |Log^2^(Fold Change)| ≥ 1 and padj < 0.05 and visualized through volcano plots. KEGG pathway enrichment analysis was conducted separately for each comparison. The results (Figure 2c,d) showed that the DEGs in the HG vs. control group were primarily enriched in glycolysis/gluconeogenesis, NF‐kappa B signaling pathway, N‐glycan biosynthesis, carbon metabolism, AMPK signaling pathway, Pl3K‐Akt signaling pathway, FoxO signaling pathway, steroid biosynthesis, MAPK signaling pathway and ferroptosis signaling pathway, whereas those in the HG + Cur vs. HG group were mostly concentrated in NLR signaling pathway, oxidative phosphorylation, focal adhesion, TNF signaling pathway, ferroptosis, TGF‐beta signaling pathway, p53 signaling pathway, celular senescence, mTOR signaling pathway, and cell cycle pathway. These findings suggest that the convergence of these pathways reveals ferroptosis as their principal common node. Cur may mitigate DKD by inhibiting ferroptosis.

Figure 2Effects of Cur intervention on transcriptomics of high‐glucose‐induced MPC‐5 cells. Cells from Control, HG, and HG + H − Cur groups were collected for transcriptomic analysis. Differentially expressed genes (DEGs) for HG versus Control and HG‐Cur versus HG were screened using |Log₂(Fold Change)| ≥ 1 and padj ≤ 0.05. (a) DEGs for HG versus Control. (b) DEGs for HG‐Cur versus HG. (c) KEGG pathway enrichment analysis of DEGs for HG versus Control. (d) KEGG pathway enrichment analysis of DEGs for HG‐Cur versus HG. *n* = 3 biologically independent samples per group.

**Figure:**
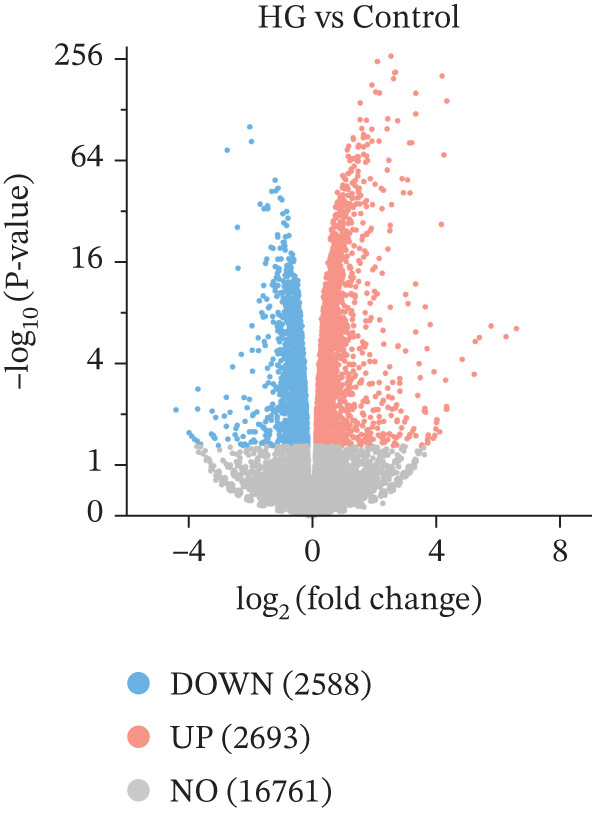
(a)

**Figure:**
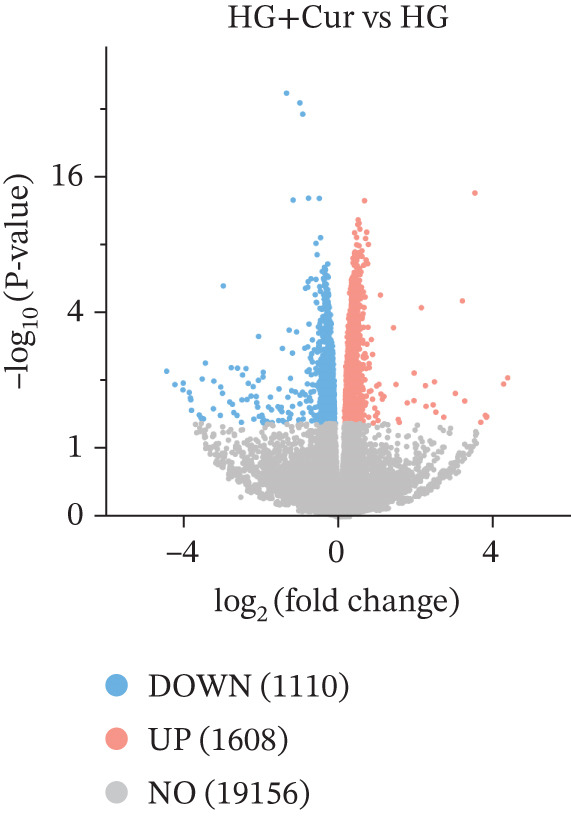
(b)

**Figure:**
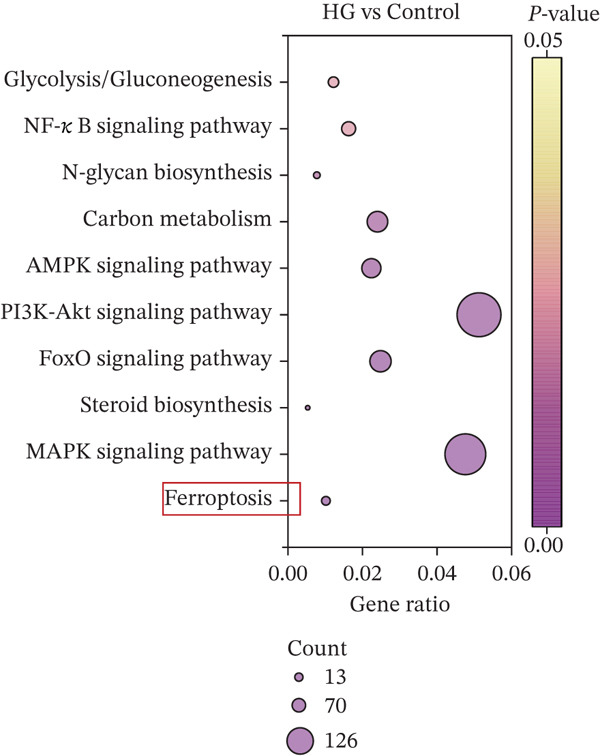
(c)

**Figure:**
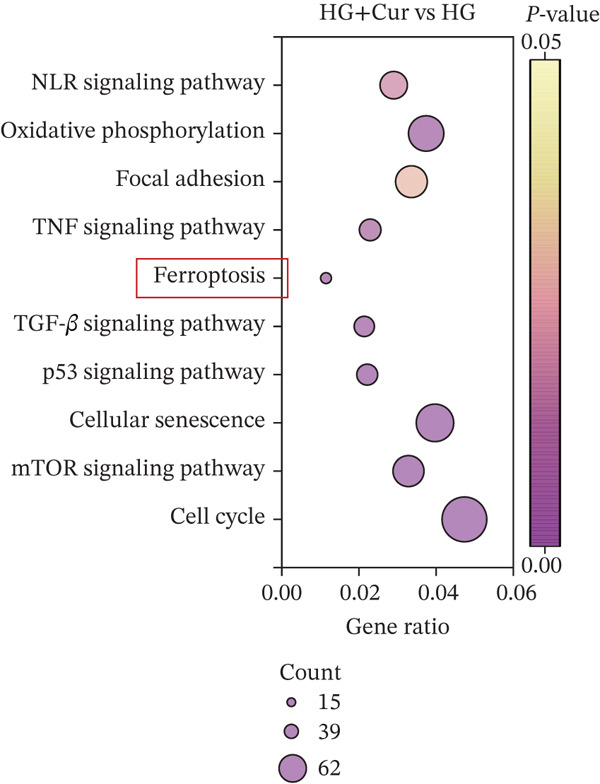
(d)

### 3.3. Cur Inhibits High–Glucose‐Induced Ferroptosis in MPC‐5 Cells

Given the strong association between transcriptomic findings and ferroptotic phenotypes, we further examined Cur′s role in inhibiting ferroptosis in the high–glucose‐induced MPC‐5 cell injury model. As shown in Figure 3a, total iron content was significantly elevated in MPC‐5 cells treated with high glucose (*p* < 0.01), indicating disturbed iron metabolism. In contrast, Cur treatment reduced total iron levels in a dose‐dependent manner (*p* < 0.05) (Figure 3a).

Figure 3Cur intervention inhibits high–glucose‐induced ferroptosis in MPC‐5 cells. (a) Total iron detection results showing that Cur intervention significantly reduces total iron content in MPC‐5 cells. *n* = 6 biologically independent samples per group. (b) Heatmap of transcriptome‐related genes indicating that Cur intervention upregulates expression of GPX4 pathway and iron metabolism, downregulates expression of the lip peroxidation. (c–h) Western blot results demonstrating that Cur intervention significantly increases protein expression of SLC3A2, SLC7A11, and GPX4, and decreases protein expression of NCOA4, ACSL4, and Trf. *n* = 3 biologically independent samples per group. When data exhibit a normal distribution with homogeneity of variance, we use ANOVA. When data exhibit a nonnormal distribution with heterogeneity of variance, we use the KW test. ∗*p* < 0.05, ∗∗*p* < 0.01. #*p* < 0.05, ##*p* < 0.01. ns: *p* > 0.05.

**Figure:**
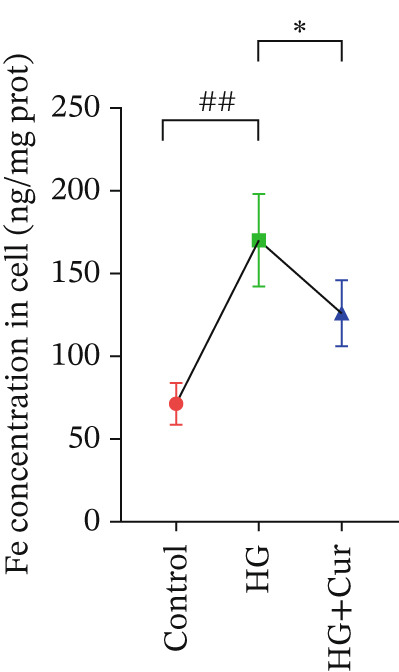
(a)

**Figure:**
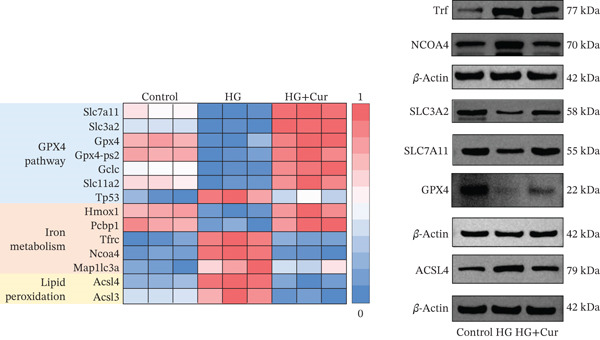
(b)

**Figure:**
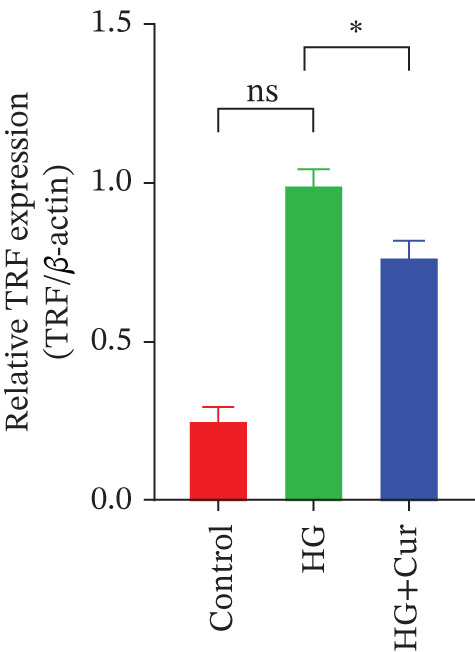
(c)

**Figure:**
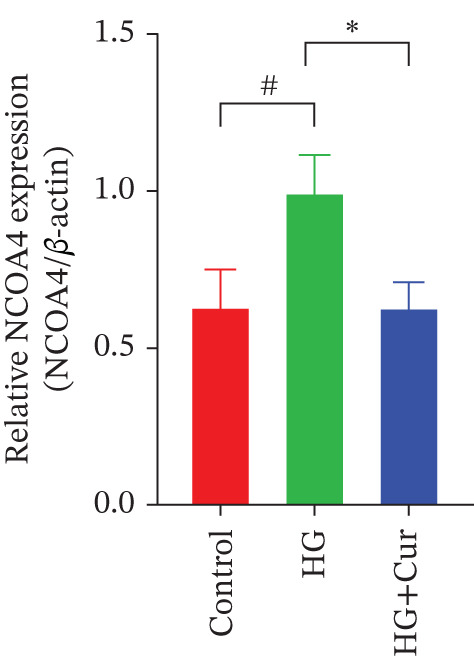
(d)

**Figure:**
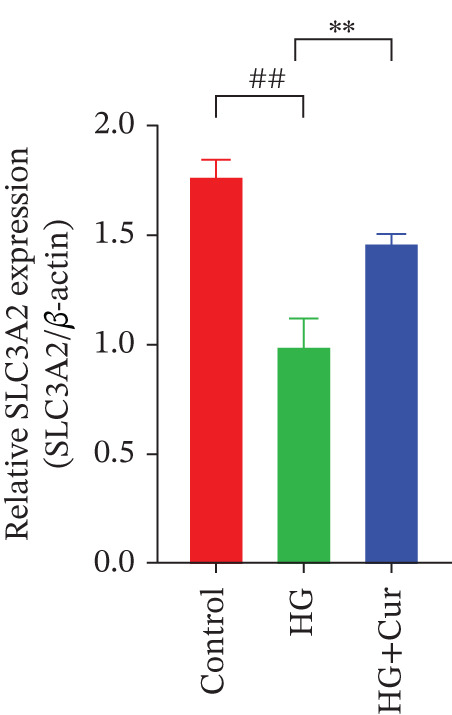
(e)

**Figure:**
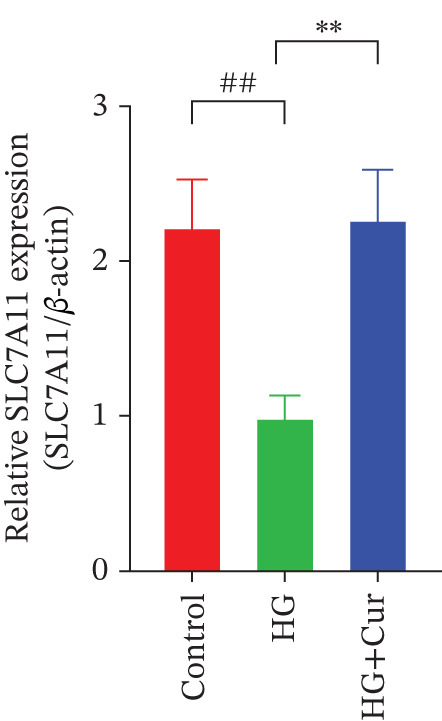
(f)

**Figure:**
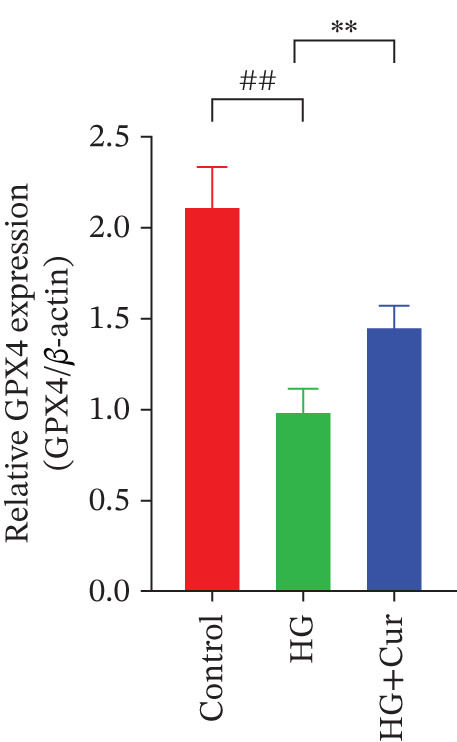
(g)

**Figure:**
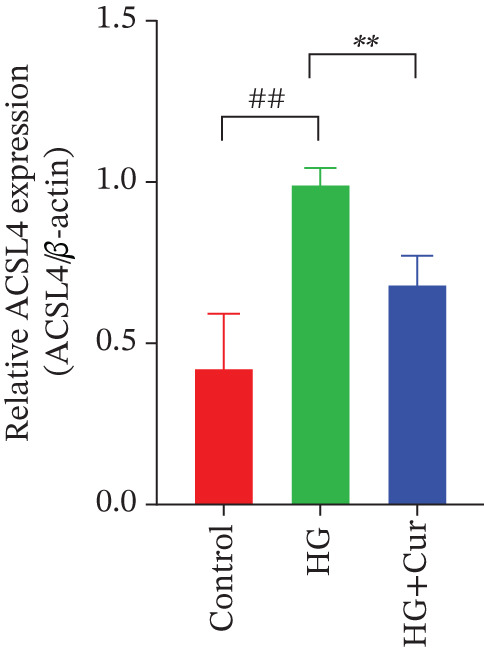
(h)

Transcriptomic analysis of ferroptosis‐related pathways (Figure 3b) revealed that high‐glucose treatment notably upregulated genes involved in iron metabolism, such as TRFc, MAP1LC3A, and NCOA4, while downregulating HMOX1 and PCBP1. In lipid peroxidation pathways, ACSL4 and ACSL3 were upregulated, and in the GPX4 pathway, high glucose led to a significant reduction in SLC7A11, SLC3A2, GPX4, GPX4‐ps2, SLC11A2, and GCLC, whereas P53 expression was upregulated. Cur treatment reversed these changes. Notably, pathways related to iron metabolism, lipid peroxidation, and the GPX4 pathway, which are associated with ferroptosis, were significantly represented. Western blot analysis (Figures 3c, 3d, 3e, 3f, 3g, and 3h) further confirmed these findings. Compared with the control group, the model group exhibited elevated levels of NCOA4, ACSL4, and TRF proteins, accompanied by decreased SLC3A2, SLC7A11, and GPX4 levels. Cur intervention alleviated these alterations, demonstrating its potential to mitigate ferroptosis in this model.

### 3.4. Curcumol Inhibits Ferroptosis Through the xCT/GPX4 Pathway

Based on previous findings, we hypothesized that Cur may exert therapeutic effects by modulating the xCT/GPX4 pathway to inhibit excessive ferroptosis activation.GPX4, a key upstream regulator of ferroptosis, plays a pivotal role in this process. To validate the antiferroptotic effects of Cur, we used the ferroptosis agonist RLS3, a covalent inhibitor of GPX4, and ferrostatin‐1 (Fer‐1) as control treatments.

RSL3 is a ferroptosis agonist. Chemical proteomics approaches have identified the antioxidant defense enzyme GPX4 as the direct drug target of RSL3 [22]. Fer‐1 is a ferroptosis inhibitor that effectively suppresses the excessive generation of ROS, inhibits ferroptosis by restoring the expression levels of ferritin and GPX4, thereby reducing lipid peroxidation [23].

**Figure 4:**
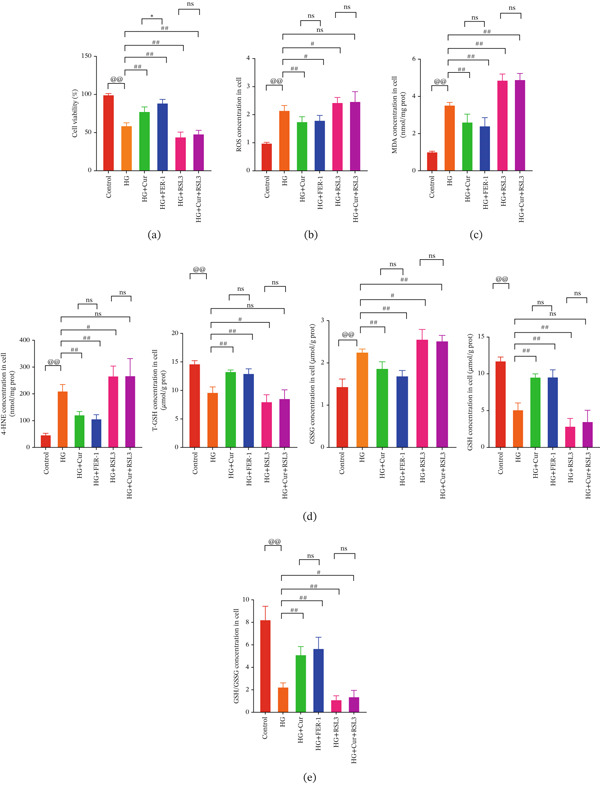
Cur inhibits ferroptosis through the Lipid Oxidation. We used RLS3 and Fer‐1 to investigate whether Cur exerts antiferroptotic effects by regulating the degree of cellular lipid oxidation. (a) MTT assay showing that RLS3 attenuates the improving effect of Cur on the viability of high–glucose‐induced MPC‐5 cells.(b–e) RLS3 treatment abolishes the reducing effect of Cur on total iron, ROS (b), MDA (d), and 4‐HNE levels. (e) In the high‐glucose‐induced MPC‐5 cells, RLS3 treatment eliminates the enhancing effect of Cur on the GSH/GSSG ratio in high–glucose‐induced MPC‐5 cells. *n* = 6 biologically independent samples per group. When data exhibit a normal distribution with homogeneity of variance, we use ANOVA. When data exhibit a nonnormal distribution with heterogeneity of variance, we use the KW test. ∗*p* < 0.05, ∗∗*p* < 0.01.#*p* < 0.05, ##*p* < 0.01. ns: *p* > 0.05.

Both Cur and Fer‐1 significantly improved MPC‐5 cell viability (*p* < 0.01), confirming their cytoprotective roles in inhibiting ferroptosis (Figure 4a). Following treatment, total iron content in the cells decreased (*p* < 0.05) (Figure 5a), indicating a reduction in iron accumulation. Additionally, levels of lipid peroxidation markers (Figures 4b, 4c, 4d, and 4e)—ROS (*p* < 0.01; *p* < 0.05), MDA (*p* < 0.01), and 4‐HNE—were significantly lower (*p* < 0.01), suggesting suppression of lipid peroxidation. Furthermore, the levels of reduced GSH and the GSH/GSSG ratio were notably elevated (*p* < 0.01), reflecting an improvement in the oxidative stress status.

Figure 5Cur inhibits ferroptosis through the xCT/GPX4 pathway. We used RLS3 and Fer‐1 to investigate whether Cur exerts antiferroptotic effects by regulating the xCT/GPX4 pathway. (a) Total iron detection results showed that the regulatory effect of Cur was weakened after RLS3 treatment. *n* = 6 biologically independent samples per group. (b–g) Western blot results demonstrating that the regulatory effect of Cur on the expression of xCT/GPX4 pathway proteins and ferroptosis phenotypic proteins was weakened after RLS3 treatment. *n* = 3 biologically independent samples per group. When data exhibit a normal distribution with homogeneity of variance, we use ANOVA. When data exhibit a nonnormal distribution with heterogeneity of variance, we use the KW test. @*p* < 0.05, @@*p* < 0.01.#*p* < 0.05, ##*p* < 0.01. ns: *p* > 0.05.

**Figure:**
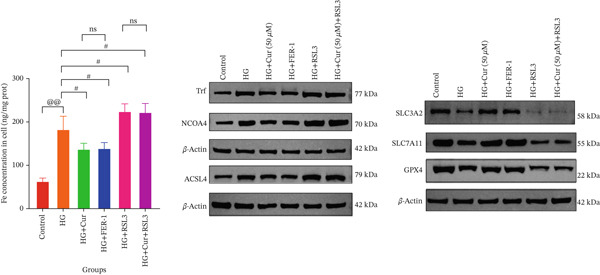
(a)

**Figure:**
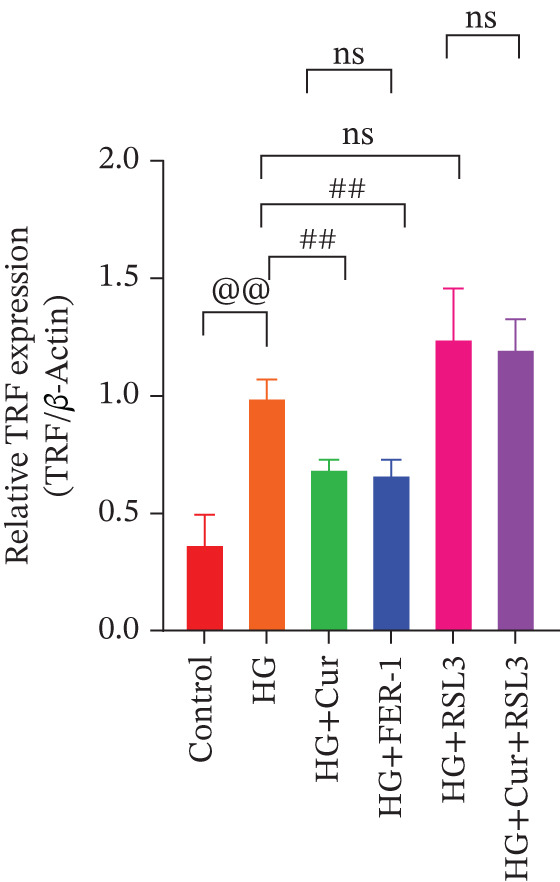
(b)

**Figure:**
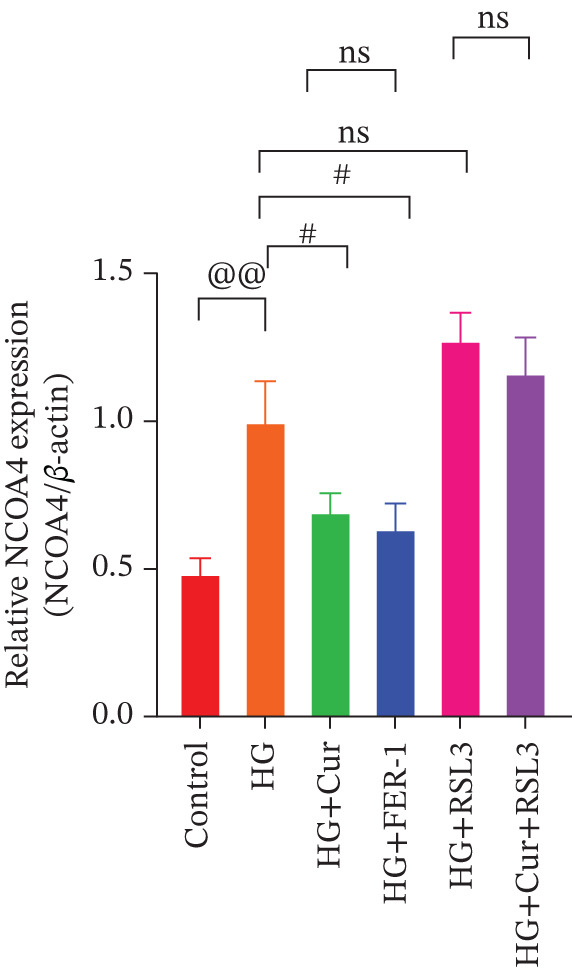
(c)

**Figure:**
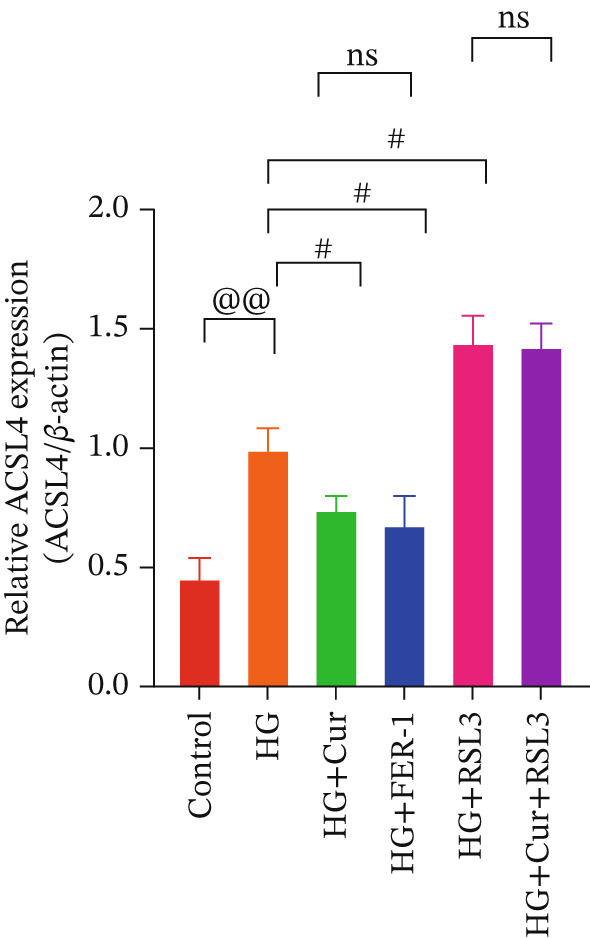
(d)

**Figure:**
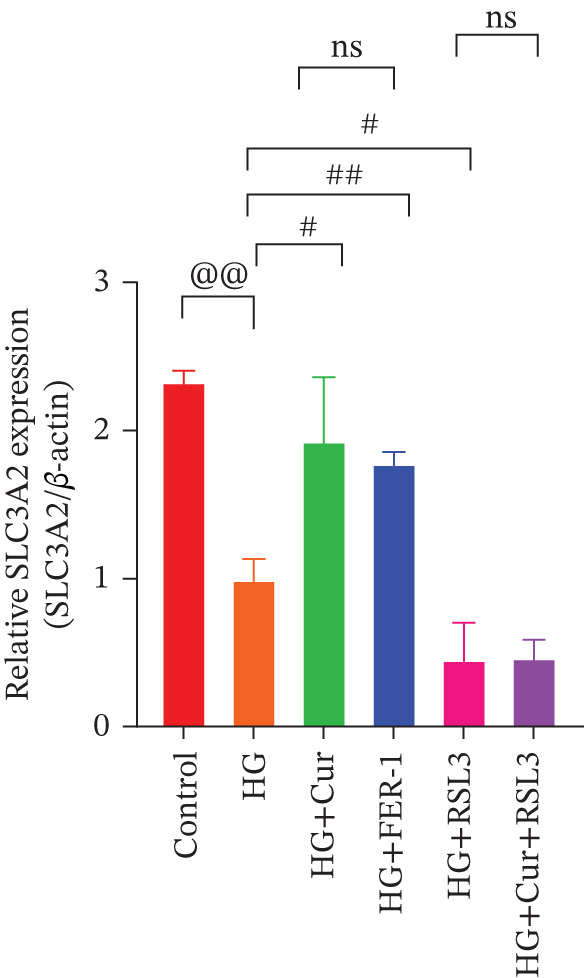
(e)

**Figure:**
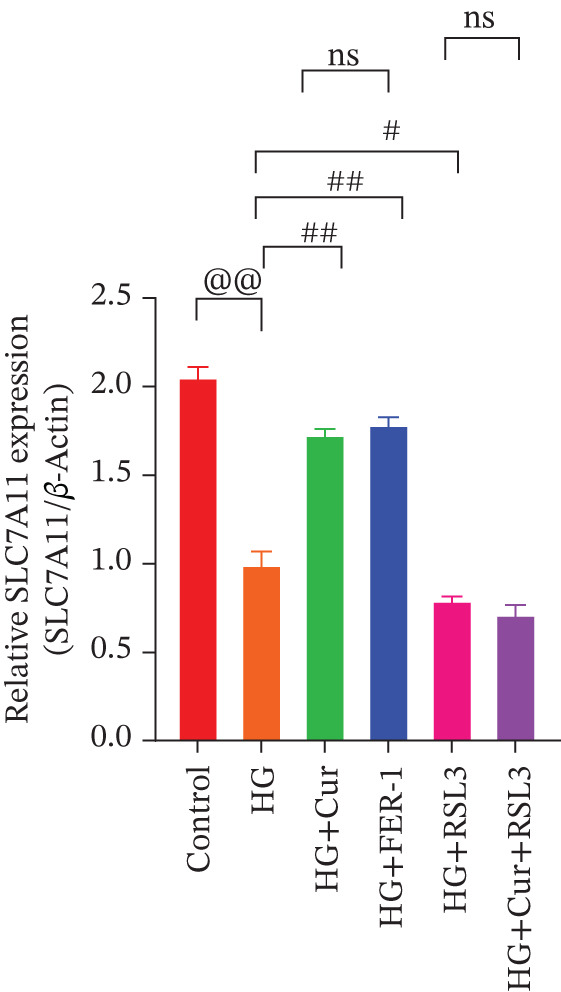
(f)

**Figure:**
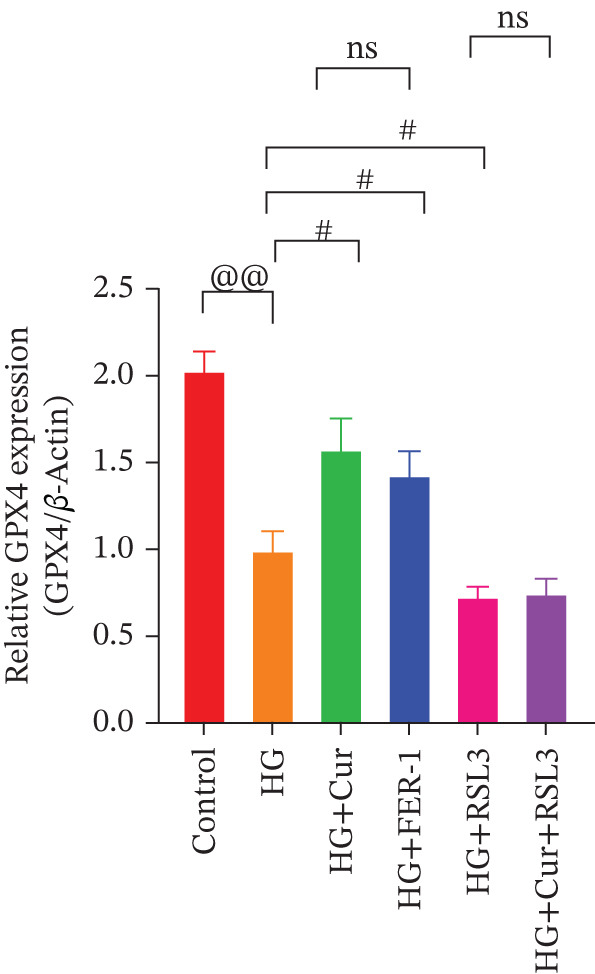
(g)

While Fer‐1 demonstrated a more pronounced effect on cell viability compared with Cur (*p* < 0.05) (Figure 4a), there were no significant differences between the two in terms of regulating iron metabolism, inhibiting lipid peroxidation, and improving oxidative stress markers (*p* > 0.05) (Figures 4b, 4c, 4d, and 4e).

The ferroptosis activator RLS3, however, completely negated the protective effects of Cur. RLS3 not only inhibited the Cur‐induced increase in cell viability(*p* < 0.01)(Figure 4a), but also exacerbated iron deposition, as evidenced by elevated total iron content(*p* < 0.05)(Figure 5a). It further promoted lipid peroxidation, indicated by increased ROS, MDA, and 4‐HNE levels(*p* < 0.05；*p* < 0.01；*p* < 0.05)(Figures 4b, 4c, and 4d), and impaired antioxidant defense, shown by a reduction in GSH content and the GSH/GSSG ratio(*p* < 0.01)(Figure 4e). Importantly, no significant differences were observed between the RLS3‐alone group and the RLS3 + Cur group for any of the measured indicators(*p* > 0.05)(Figures 4b, 4c, 4d, and 4e), suggesting that RLS3 effectively counteracted Cur′s therapeutic effects.

Compared with the normal control group, the model group exhibited a significant increase in the expression of ferroptosis‐related proteins including transferrin receptor (TRF) and nuclear receptor coactivator 4 (NCOA4) (*p* < 0.01) (Figure 5b,c), alongside a marked rise in the lipid peroxidation marker, acyl‐CoA synthetase long‐chain family member 4 (ACSL4) (*p* < 0.01) (Figure 5d). In contrast, the expression of key components of the xCT/GPX4 pathway, such as SLC3A2, SLC7A11, and GPX4, was significantly reduced (*p* < 0.01) (Figures 5e, 5f, and 5g). These results indicate that ferroptosis was activated, lipid peroxidation was aggravated, and the function of the GSH‐GPX4 antioxidant system was impaired in the model group.

Treatment with either or the ferroptosis inhibitor Fer‐1 notably reversed the pathological changes induced by the model. This was evidenced by decreased expression levels of TRF, NCOA4, and ACSL4 proteins (*p* < 0.01; *p* < 0.05; *p* < 0.05) (Figures 5b, 5c, and 5d), alongside upregulation of SLC3A2 (*p* < 0.05, *p* < 0.01), SLC7A11 (*p* < 0.01), and GPX4 proteins (*p* < 0.05) (Figures 5b, 5c, 5d, 5e, 5f and 5g). There were no significant differences between the effects of Cur and Fer‐1 (*p* > 0.05) (Figures 5b, 5c, 5d, 5e, 5f and 5g), suggesting that both interventions produced comparable therapeutic outcomes.

In contrast, treatment with the ferroptosis activator RLS3 exacerbated the injury phenotype in the model group. The levels of Tfr and NCOA4 showed no significant difference compared with the model group (*p* > 0.05) (Figure 5b,c). The protein expression of ACSL4 was increased (*p* < 0.05) (Figure 5d), whereas the expressions of SLC3A2, SLC7A11, and GPX4 were decreased (*p* < 0.01; *p* < 0.01; *p* < 0.05) (Figures 5e, 5f, and 5g). Importantly, RLS3 completely counteracted the protective effects of curcumol, with no significant differences observed in any of the indicators between the RLS3 alone group and the RLS3 + curcumol group(*p* > 0.05) (Figures 5b, 5c, 5d, 5e, 5f, and 5g).

### 3.5. Cur Intervention Ameliorates Renal Injury in DKD Mice

**Figure 6:**
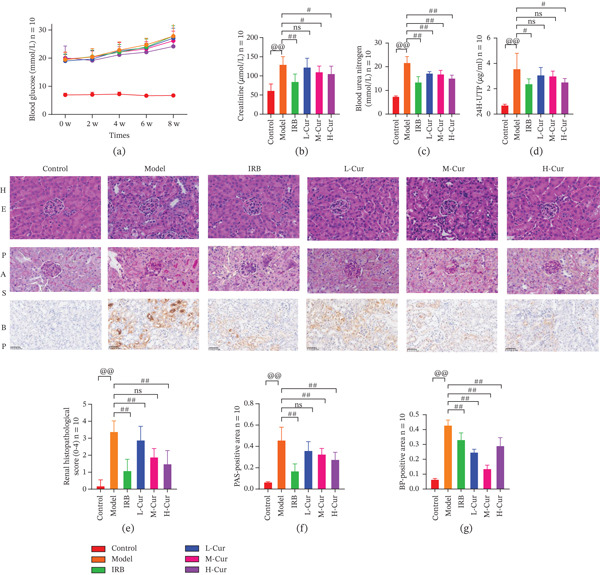
Cur intervention ameliorates renal injury in DKD mice. The DKD mouse model was established, and Cur intervention was applied to evaluate its in vivo efficacy. (a) Cur intervention slightly reduces fasting blood glucose (FBG) in DKD mice. (b–d) Cur intervention decreases levels of serum Cr, blood urea nitrogen (BUN), and 24‐h urinary total protein (24 h‐UTP) in DKD mice. (e) Cur intervention lowers the renal injury score in DKD mice (HE staining). (f) Cur intervention alleviates renal glycogen deposition in DKD mice (PAS staining). (g) Cur intervention reduces renal iron deposition in DKD mice (BP staining). *n* = 10 biologically independent samples per group. When data exhibit a normal distribution with homogeneity of variance, we use ANOVA. When data exhibit a nonnormal distribution with heterogeneity of variance, we use the KW test. @*p* < 0.05, @@*p* < 0.01. #*p* < 0.05, ##*p* > 0.05.

Building on the in vitro findings, we next established a DKD mouse model to assess the in vivo efficacy of Cur. Fasting blood glucose (FBG) levels, as shown in Figure 6a, were significantly elevated in the DKD group compared with the control group. However, both the IRB and H‐Cur groups exhibited a modest reduction in FBG levels.

Renal function indicators (Figures 6b, 6c, and 6d) revealed that the levels of serum Cr, BUN, and 24‐h urinary protein were significantly elevated in the DKD group compared with the control group (*p* < 0.01). Both IRB and Cur interventions improved these indicators in a dose‐dependent manner, with higher concentrations of Cur showing more pronounced effects. Nonetheless, the therapeutic impact of the Cur treatments was less than that of IRB.

Renal histopathological analysis (Figures 6e, 6f, and 6g) confirmed that the DKD group exhibited significantly higher renal injury scores compared with the control group. Key pathological features included glomerular hypertrophy, thickened basement membranes, increased extracellular matrix deposition, and elevated iron accumulation. Intervention with IRB and Cur effectively mitigated these pathological alterations, highlighting the potential of Cur in alleviating DKD‐related renal damage.

### 3.6. Cur Exerts Antiferroptotic Effects in DKD Mice

**Figure 7:**
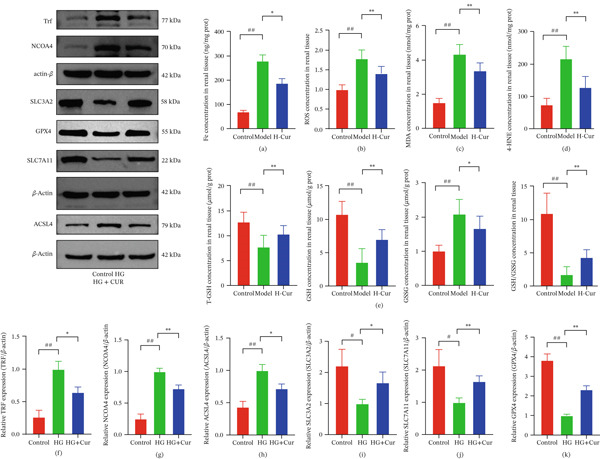
Cur inhibits renal ferroptosis in DKD mice through the xCT/GPX4 pathway. (a) Intervention with the high‐dose Cur group significantly reduces total iron level in renal tissues of DKD mice. (b–d) High‐dose Cur intervention markedly decreases levels of ROS (b), MDA (c), and 4‐HNE (d) in renal tissues. (e) High‐dose Cur intervention significantly upregulates the GSH/GSSG ratio in renal tissues. (f–k) High‐dose Cur intervention significantly increases protein expression of SLC3A2, SLC7A11, and GPX4 in renal tissues, and reduces protein expression of NCOA4, ACSL4, and Trf. *n* = 10 biologically independent samples for a–*d*, *n* = 3 biologically independent samples for f–k. When data exhibit a normal distribution with homogeneity of variance, we use ANOVA. When data exhibit a nonnormal distribution with heterogeneity of variance, we use the KW test. ∗*p* < 0.05, ∗∗*p* < 0.01. #*p* < 0.05, ##*p* < 0.01.

Subsequently, we further investigated the antiferroptotic effects of Cur in vivo. As shown in Figures 7a, 7b, 7c, 7d, and 7e, results from kit‐based assays revealed that treatment with H‐Cur significantly reduced total iron (*p* < 0.01), ROS (*p* < 0.01), MDA (*p* < 0.01), and 4‐HNE (*p* < 0.01) levels in renal tissue homogenates from DKD mice. Additionally, H‐Cur treatment increased the GSH/GSSG ratio (*p* < 0.01). Western blot analysis (Figures 7f, 7g, 7h, 7i, 7j, and 7k) indicated that H‐Cur upregulated the expression of SLC3A2, SLC7A11, and GPX4 proteins in the xCT/GPX4 pathway while downregulating the expression of TRF, NCOA4, and ACSL4 proteins. These findings align with the in vitro results.

## 4. Discussion

DKD has become one of the most significant public health problems, seriously threatening the health of the nation′s population. It not only damages health and diminishes quality of life but also increases the substantial burden on patients′ families. Addressing this challenge proactively will alleviate pressure on the healthcare system and promote the long‐term sustainable development of public health.

In our in vitro experiments, we established a high–glucose‐induced MPC‐5 cell injury model to evaluate the therapeutic potential of curcumol for DKD. This model mimics high–glucose‐induced podocyte damage, providing a reliable platform for identifying safe drug concentrations and exploring the underlying mechanisms [24] Our results demonstrated that curcumol significantly enhanced the viability of MPC‐5 cells exposed to high–glucose toxicity, suggesting that curcumol mitigates podocyte damage caused by hyperglycemia.

ROS are a collective term for the entire family of oxidants derived from molecular oxygen. These species participate in redox reactions and modify biological macromolecules, thereby contributing to redox signal transduction and reproductive functions [25]. The accumulation of ROS is a key feature of ferroptosis [6]. Lipid ROS specifically target polyunsaturated fatty acids (PUFAs) within cell membranes, initiating a chain reaction of lipid peroxidation. This process generates toxic byproducts, such as MDA and 4‐HNE, ultimately leading to lytic cell death [26]. The increase in ROS, MDA, and 4‐HNE levels, which reflect the extent of lipid peroxidation, serves as a critical indicator of ferroptosis. Measuring ROS, MDA, and 4‐HNE levels in cells or tissues provides an indirect assessment of the extent of ferroptosis.

GSH is the main antioxidant in mammalian cells, including reduced GSH and oxidized GSSG [27]. As the substrate for glutathione peroxidase (GPx) and glutathione S‐transferase (GST), GSH cleans up harmful substances like free radicals and lipid peroxides, converting them into fatty acids and water, during which process it is itself oxidized to form GSSG [28, 29]. The intracellular level of GSH is dynamically regulated by its synthesis, consumption, and efflux. Due to diminished synthesis, increased consumption, or enhanced efflux, GSH is depleted rapidly. This depletion impairs the cell′s ability to eliminate lipid peroxides, ultimately triggering ferroptosis [30].

High glucose treatment led to a significant increase in the accumulation of ROS, MDA, and 4‐HNE in MPC‐5 cells, whereas the GSH/GSSG ratio decreased. Combined with transcriptomic consequences, high‐glucose treatment was shown to induce ferroptosis. With Cur treatment, these effects were effectively counteracted, primarily by reducing the levels of oxidative markers and restoring the GSH/GSSG balance. Transcriptomic evidence confirmed the inhibitory role of Cur in high glucose‐induced ferroptosis.

A significant increase in total iron levels is a clear indicator of iron deposition. Although it cannot distinguish between iron valence states, it is a stable indicator that, as total iron content increases, the availability of substrates for the Fenton reaction also increases. The valence state of intracellular iron is dynamic, with Fe^2+^ being particularly unstable, as it is readily oxidized or bound by proteins.

TFRC, a homodimeric protein located on the cell surface, is a key marker of ferroptosis. When combined with TF, which contains Fe3+, the resulting TF‐TFRC complex mediates iron uptake endocytosis [31]. NCOA4, a selective receptor, facilitates the autophagic degradation of ferritin through the process of ferritinophagy. Since ferritinophagy plays a crucial role in maintaining iron homeostasis, it supports iron‐dependent physiological processes [32].

MAP1LC3A (microtubule‐associated protein 1 light chain 3 alpha), an autophagosomal marker, participates in the formation and maturation of autophagosomes. During the process of ferroptosis, autophagy can be activated to remove damaged organelles or degrade proteins associated with ferroptosis. For instance, MAP1LC3A and GPX4 track the autophagic degradation of GPX4. This observation suggests that autophagy may facilitate GPX4 degradation, which promotes ferroptosis [33]. In the studies of glioma, the level of MAP1LC3A is considered to be combined with the susceptibility to ferroptosis. Its downregulation may be associated with increased resistance of tumor cells to ferroptosis [34].

Our results revealed that under high‐glucose stress, it is the manifestations of significant disruption in cellular iron metabolism that the expression of Tfr, NCOA4, and MAP1LC3A is significantly increased, which leads to elevated intracellular iron uptake and enhanced ferritinophagy. This finding is consistent with the observed increase in total cellular iron levels.

PCBP1, a negative regulator of ferroptosis, can be knocked down to result in the accumulation of intracellular Fe^2+^ and promote ferritinophagy, thereby enhancing cellular susceptibility to ferroptosis. Meanwhile, it led to an increase in the levels of 4‐HNE, MDA, and several other markers while reducing lipid peroxidation. PCBP1 helps maintain intracellular iron homeostasis and lipid metabolic balance, thereby protecting cells from ferroptosis [35]. Therefore, under high‐glucose conditions, the observed downregulation of PCBP1 expression contributes to the enhanced activation of ferroptosis.

ACSL4 is an enzyme responsible for converting fatty acids into fatty acyl‐CoA esters, playing a key role in regulating lipid biosynthesis. In the context of acute kidney injury (AKI), ACSL4 expression often correlates positively with injury severity, positioning it as a potential target for preventing and treating AKI by inhibiting ferroptosis [36]. In our study, elevated ACSL4 expression enhanced lipid peroxidation of PUFAs, resulting in increased levels of oxidative stress markers such as ROS, MDA, and 4‐HNE. Furthermore, inhibition of the SLC7A11‐GSH‐GPX4 antioxidant system is a central mechanism in ferroptosis [37].

The xCT/GPX4 axis constitutes a central pathway in ferroptosis, whose inhibition is a key driver of this cell death process [38]. The SLC7A11‐GSH‐GPX4 cascade forms the core of this pathway. System Xc^−^, a heterodimer composed of SLC7A11 and SLC3A2, facilitates cystine uptake to support glutathione (GSH) synthesis. GPX4 subsequently utilizes GSH to reduce lipid peroxides and maintain membrane integrity. However, this tightly regulated system is vulnerable: SLC7A11 expression is subject to complex regulatory controls, and GPX4 can be inactivated through degradative pathways such as autophagy. Consequently, the downregulation or functional impairment of key components like SLC7A11 and GPX4 directly leads to GSH depletion and GPX4 inactivation, triggering lethal lipid peroxide accumulation and ultimately initiating ferroptosis [39, 40].

GCLC (glutamate‐cysteine ligase catalytic subunit) is the core effector molecule in ferroptosis. It is its core function that inhibits iron‐dependent lipid peroxidation by mediating GSH synthesis, averting cells from ferroptosis [41].

There are some genes that have been demonstrated to exert dual regulatory roles in ferroptosis:

HMOX1 (heme oxygenase 1), a central enzyme in heme catabolism, releases (Fe^2+^). Through the Fenton reaction with H_2_O_2_, Fe^2+^ catalyzes the production of hydroxyl radicals (OH) and induces lipid peroxidation, leading to phospholipid hydroperoxide (PLOOH) formation. The breakdown of PLOOHs generates toxic aldehydes such as 4‐HNE and MDA, which are critical mediators of ferroptosis [42]. On the contrary, in glioblastoma, HMOX1 activation has been shown to attenuate ferroptosis by reducing lipid peroxidation, ROS production, and intracellular Fe^2+^ levels following irradiation [43].

ACSL3 inhibits ferroptosis by activating monounsaturated fatty acids (MUFAs) and reducing oxidisable polyunsaturated fatty acid phospholipids (PUFA‐PLs) in cell membranes [44]. However, previous studies have demonstrated that ACSL3 can increase ferroptosis sensitivity by activating PUFAs [45].

P53, a transcription factor capable of regulating multiple targets, plays a diametrically opposed role in sensitivity to iron deficiency. On the one hand, it reduces ferroptosis susceptibility by stabilizing intracellular glutathione (GSH) levels via system Xc−. On the other hand, it enhances ferroptosis sensitivity by inducing cell cycle arrest and remodeling the composition of cell membrane lipids via the GPX4 pathway. In contrast, the role of the xCT/GPX4 pathway in ferroptosis is clear and unidirectional [46].

In contrast, the role of the xCT/GPX4 pathway in ferroptosis is clear and unidirectional.

At the genetic level, high glucose significantly reduces the expression of SLC7A11, SLC3A2, and GPX4, which are key components of system Xc−. Cur intervention effectively reverses the expression of these genes. At the protein level, Western blot results further confirm that high glucose decreases the protein expression of SLC3A2, SLC7A11, and GPX4. In contrast, Cur treatment restores the expression of these proteins. These consistent changes from mRNA to protein specifically indicate that the xCT/GPX4 pathway is a crucial downstream component in the functioning of Cur, rather than a mere coincidence. Therefore, we speculate that Cur may inhibit cell ferroptosis through the xCT/GPX4 pathway.

Many previous studies have demonstrated that ferroptosis is a significant mechanism of renal tubular cell death under diabetic conditions. The downregulation of xCT and GPX4 expression reduces glutathione synthesis and increases lipid peroxidation, ultimately inducing ferroptosis [47].

Our research results indicate that Cur treatment may significantly restore cell vitality; however, it cannot reverse the cytotoxicity induced by the specific GPX4 inhibitor RSL3, suggesting that Cur exerts its protective effects via the xCT/GPX4 pathway. Cur reduced the levels of total iron, ROS, MDA, and 4‐HNE while simultaneously increasing GSH content. These changes indicate that Cur prevents ferroptosis by restoring lipid redox balance. Integrating transcriptomic data with experimental findings, we observed that curcumol treatment significantly alleviates the pathological changes associated with ferroptosis. These effects include (1) downregulation of TRFC and NCOA4 expression, which reduces iron uptake and ferritinophagy, thereby decreasing intracellular iron accumulation; (2) inhibition of ACSL4‐mediated lipid peroxidation, resulting in reduced levels of ROS, MDA, and 4‐HNE; (3) upregulation of SLC3A2, SLC7A11, and GPX4 expression, thereby restoring the function of the GSH‐GPX4 antioxidant system.

Collectively, these mechanisms describe how curcumol inhibits ferroptosis through multiple pathways, including modulation of iron metabolism, suppression of lipid peroxidation, and enhancement of antioxidant defense.

In our in vivo experiments, we established a DKD mouse model by combining HFD feeding with intraperitoneal streptozotocin (STZ) injection. This model replicates the progression of exhibiting pathological features such as glomerulosclerosis and tubulointerstitial fibrosis that closely resemble those observed in human DKD. Therefore, it provides an ideal platform for investigating the mechanisms underlying early intervention in DKD [48, 49].

Our results demonstrated that Cur treatment significantly improved renal function in mice. It marked reductions in Scr, BUN, and 24‐h urinary protein levels. These findings indicate enhanced glomerular filtration and tubular reabsorption capacity. Curcumol treatment not only alleviated renal pathological injury but also significantly inhibited renal iron deposition, suggesting its link to ferroptosis inhibition in DKD. Biomolecular assays revealed that curcumol markedly decreased lipid peroxidation levels and regulated the expression of iron metabolism proteins, indicating its capacity to mitigate oxidative stress and cellular iron overload. Furthermore, curcumol intervention significantly modulated the key ferroptosis‐regulating xCT/GPX4 axis. It is evident that upregulated xCT expression and restored GPX4 protein levels. This activation enhanced the cellular capacity to eliminate phospholipid peroxides, thereby suppressing ferroptosis. The close concordance between these in vivo findings and our cellular experiments underscores the consistency of curcumol′s mechanism of action across experimental models.

## 5. Conclusion

**Figure 8:**
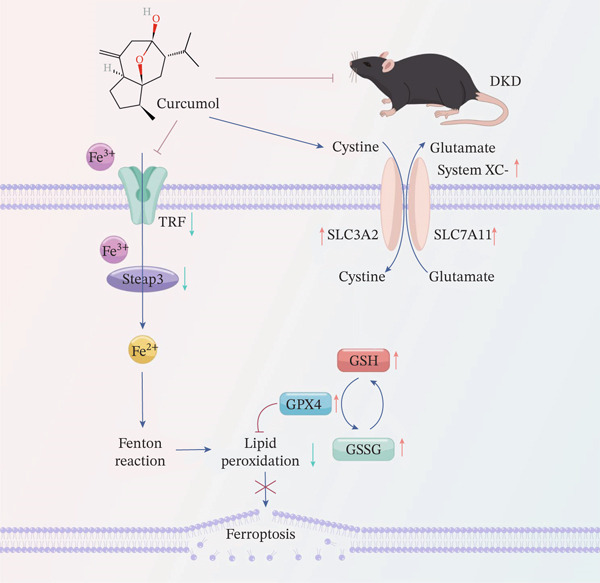
Cur exerts therapeutic effects on DKD by activating the xCT/GPX4 pathway to inhibit podocyte ferroptosis.

This study confirms that ferroptosis induced by high glucose concentration is a critical factor promoting the progression of DKD. By ameliorating dysregulated cellular iron metabolism and suppressing lipid peroxidation, curcumol (Cur) effectively inhibits ferroptosis and protects podocytes. Mechanistically, Cur upregulates the xCT/GPX4 pathway, which restores endogenous antioxidant capacity. This enhancement of GPX4‐mediated clearance of lipid peroxides contributes to its protective effects. Based on these findings, we propose a hypothesis, as shown in Figure 8, outlining the role of Cur in mitigating ferroptosis during DKD progression. We also demonstrate that the therapeutic effects of H‐Cur are comparable with those of IRB, suggesting that curcumol holds promise as a potential therapeutic candidate for DKD. Our study not only deepens the understanding of DKD pathogenesis but also provides important experimental evidence supporting the development of Cur as a therapeutic agent for DKD.

Furthermore, a limitation of our study is that we have not ruled out the possibility that Cur inhibits ferroptosis through other mechanisms. This aspect requires further investigation by future scholars.

**Nomenclature**
Cur: curcumol
WB: Western blot
Fer‐1: ferroptosis inhibitor.
DKD: diabetic kidney disease
eGFR: estimated glomerular filtration rate
MRAs: mineralocorticoid receptor antagonists
GSH: glutathione, GPX4 = glutathione peroxidase 4
ROS: reactive oxygen species
DEGs: differentially expressed genes
HFD: high‐fat diet
STZ: streptozotocin
T2DM: type 2 diabetes mellitus
Twenty four h‐UTP: 24‐h urinary total protein
L‐Cur: low‐dose Cur group.
M‐Cur: medium‐dose Cur group
H‐Cur: high‐dose Cur group.
Cr: creatinine
BUN: blood urea nitrogen
HE: hematoxylin‐eosin
PAS: periodic acid‐Schiff
PB: Prussian blue
ANOVA: one‐way analysis of variance
TRFC: transferrin receptor
TF: transferrin
NCOA4: nuclear receptor coactivator 4
ACSL4: acyl‐CoA synthetase 4
AKI: acute kidney injury
GSSG: glutathione disulfide

## Ethics Statement

All procedures complied with ARRIVE guidelines. In all animal experiments, the animals were treated ethically and humanely, and the experiments were approved by Dongzhimen Hospital, Beijing University of Traditional Chinese Medicine.

## Consent

The authors have nothing to report.

## Conflicts of Interest

The authors declare no conflicts of interest.

## Author Contributions

Yue Ji, Yuqi Wu, and Jingyi Tang contributed equally to the article.

## Funding

This study was supported by the National Natural Science Foundation of China Youth Program (82405313, 82205037, 82274293, 82274441); China Postdoctoral Science Foundation (10.13039/501100002858, 2024 M760287); Dongzhimen Hospital Foundation(DZMG‐ZJXY‐23004, DZMG‐QNZX‐24005, DZMG‐LJRC0012).

## Supporting Information

Additional supporting information can be found online in the Supporting Information section.

## Supporting information


**S1** Reagents

**S2** Western Blot

**S3** Animal Experimental Ethical Inspection The ARRIVE guidelines

## Data Availability

All primary research outputs are embedded in the text, while raw records can be procured from the designated author via formal inquiry. Data are available on request
