# Supplementary material for: Curcumol Ameliorates Diabetic Nephropathy by Inhibiting Podocyte Ferroptosis Through the xCT/GPX4 Pathway
Source: J Diabetes Res. 2026 Mar 31;2026:5577736. doi: 10.1155/jdr/5577736 (PMC13140817; doi:10.1155/jdr/5577736)
Supplement: Supplementary file 1 — S1 Reagents [file JDR-2026-5577736-s001.docx]

***Supplementary Material***

**Reagents**

Curcumol (Cur), MedChem Express ([HY-N0104](https://www.medchemexpress.cn/Curcumol.html)), Fetal Bovine Serum (Gibco,2176404), DMEM/F12(Gibco,8122157), PBS(Cytiva, SH30256.01) Streptozotocin (S17049), irbesartan (S42406) were purchased from Shanghai yuanye Bio-Technology Co., Ltd. (Shanghai, China). 24h-UTP(C035-2-1), creatinine (Cr, C011-2-1) and blood urea nitrogen (BUN, C013-2-1), BCA(A045-4-2), HE staining assay kit ([D006-1-1](http://www.njjcbio.com/products.asp?id=476)), PAS (D004-1-1) were purchased from Nanjing Jiancheng Biological Engineering Institute (Nanjing, China). Prussian blue (S0174) purchased from Beijing Bioss Biotechnology Co., Ltd.(Beijing, China). Superoxide dismutase detection kit (A001-3-2) and glutathione peroxidase detection kit (A005-1-1), malondialdehyde (A003-1-2), 4-hydroxynonenal (H268-1-1), reactive oxygen (E004-1-1) detection kits are all from Nanjing Jiancheng Biological Engineering Institute (Nanjing, China). RIPA lysis buffer (PBW003W0), PMSF(PBW007W0 ), BCA assay kits (PBW011W1), electrophoresis (PBW030W1) and transferred ([PBW031W0](http://101.201.35.2/pub/pxsw/cpzx/wb/njfl/ZMY/202503/t20250313_742.html)) The ECL Plus special sensitive chemiluminescence reagent (PBW041W0) were purchased from Tianjin Perseebio Biotechnology Co., Ltd.(Beijing, China). 4–12% precast gel (LABLEAD, P41215, P00815). polyvinylidene difluoride (PVDF) membranes (Millipore, USA). Secondary antibodies (bs-0295G-HRP, 1:5000). GPX4 (67763-1-Ig, 1:4000), ACSL4 (22401-1-AP, 1:10000), CD98/SLC3A2 (15193-1-AP 1:10000 ) were all purchased from Wuhan Proteintech Biotechnology Co., Ltd. TRF1(ab1423，1:1000), NCOA4 (ab86707, 1:1000 ) were purchased from Abcam (Shanghai, China), SLC7A11(:#98051 Cell Signaling Technology 1:1000 )
