# Supplementary material for: Curcumol Ameliorates Diabetic Nephropathy by Inhibiting Podocyte Ferroptosis Through the xCT/GPX4 Pathway
Source: J Diabetes Res. 2026 Mar 31;2026:5577736. doi: 10.1155/jdr/5577736 (PMC13140817; doi:10.1155/jdr/5577736)
Supplement: Supplementary file 2 — S2 Western Blot [file JDR-2026-5577736-s002.pdf]

cell groups： C、M、M+Cur

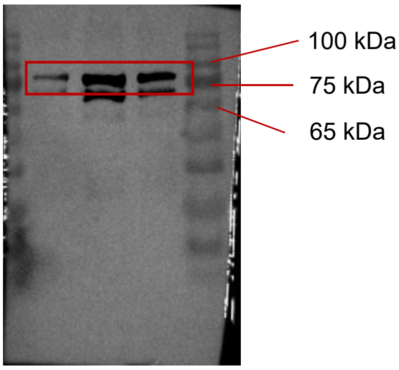

Trf 77 kDa

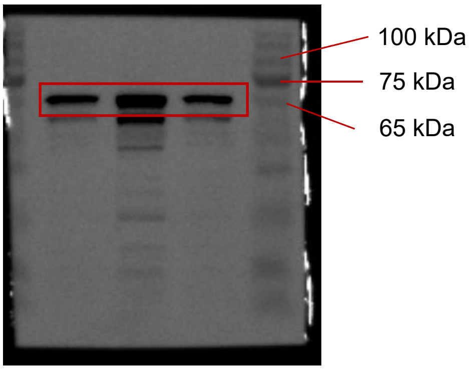

NCOA4 70 kDa

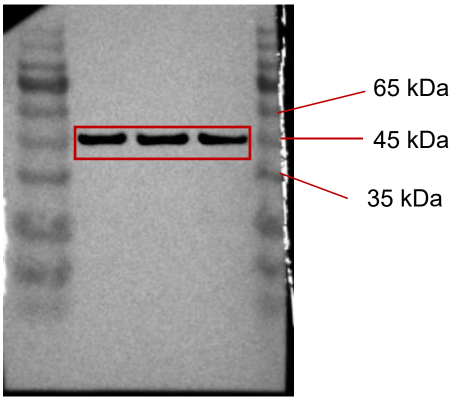

β-actin 42 kDa

cell groups： C、M、M+Cur

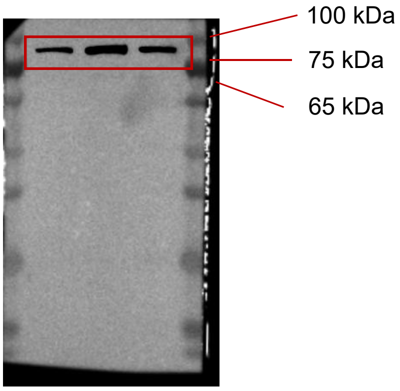

ACSL4 80 kDa

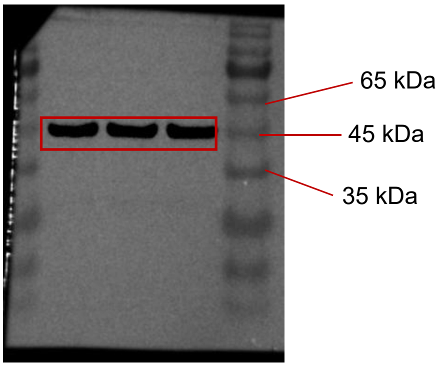

β-actin 42 kDa

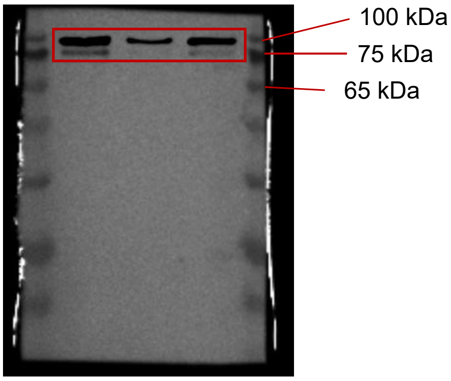

SLC3A2 80 kDa

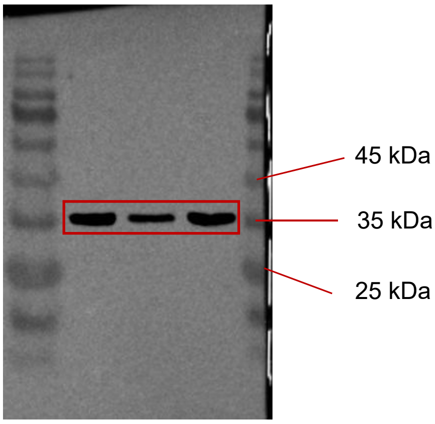

SLC7A11 35 kDa

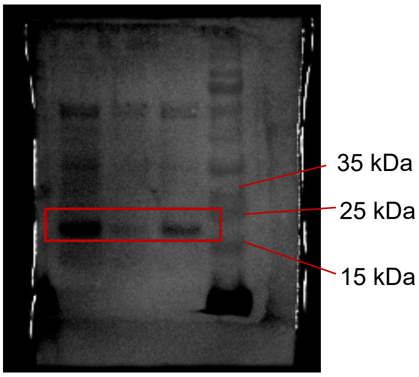

GPX4 21 kDa

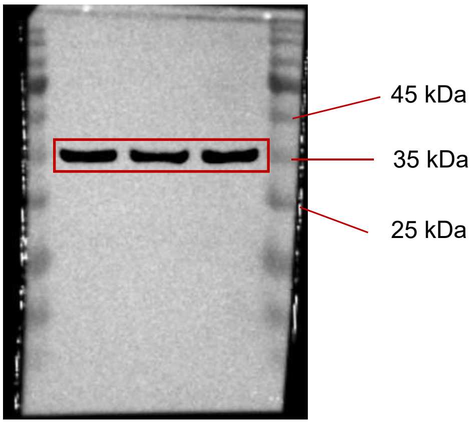

β-actin 42 kDa

cell groups： C、M、M+Cur、M+FER-1、M+RSL3、M+Cur+RSL3

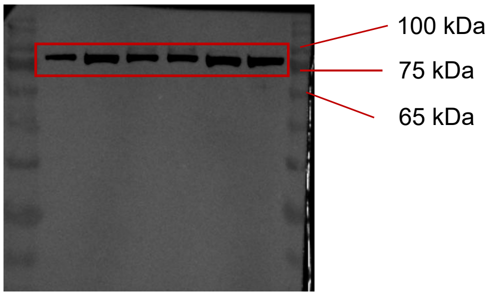

Trf 77 kDa

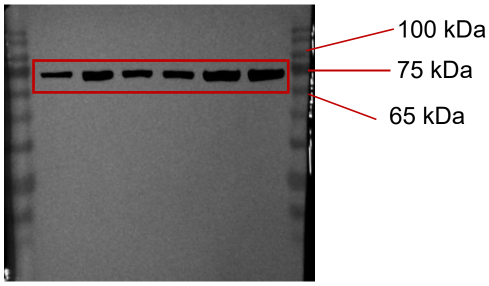

NCOA4 70 kDa

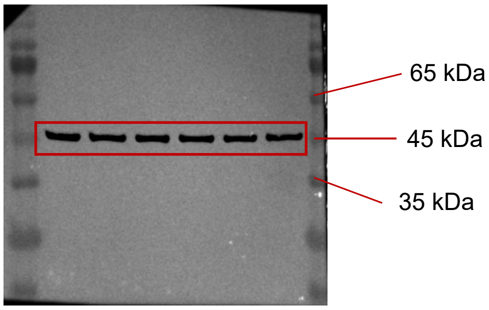

β-actin 42 kDa

cell groups：C、M、M+Cur、M+FER-1、M+RSL3、M+Cur+RSL3

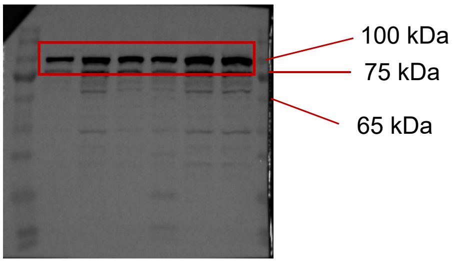

ACSL4　80 kDa

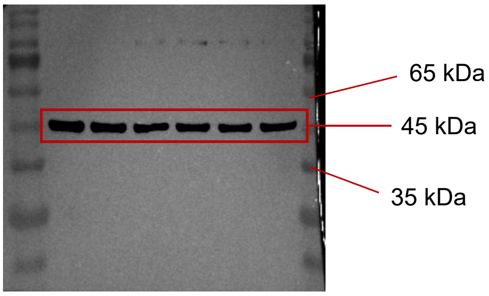

β-actin　42 kDa

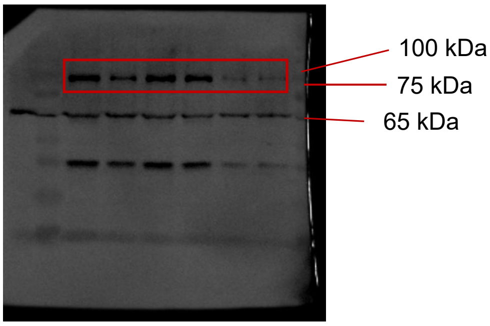

SLC3A2　80 kDa

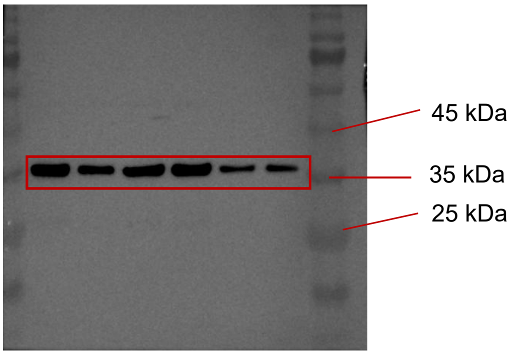

SLC7A11　35 kDa

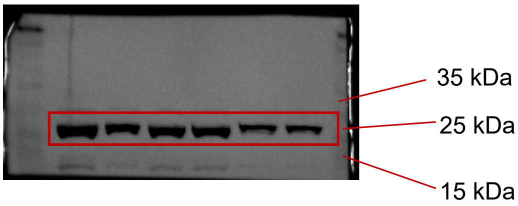

GPX4　21 kDa

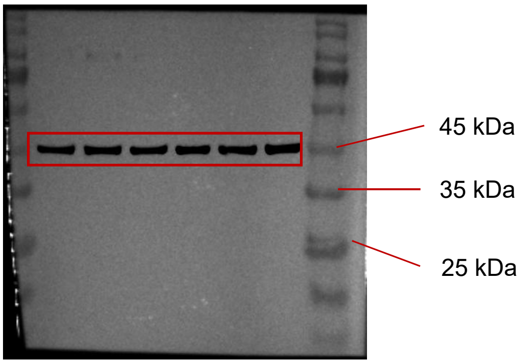

β-actin　42 kDa

kidney tssues C、M、M+Cur

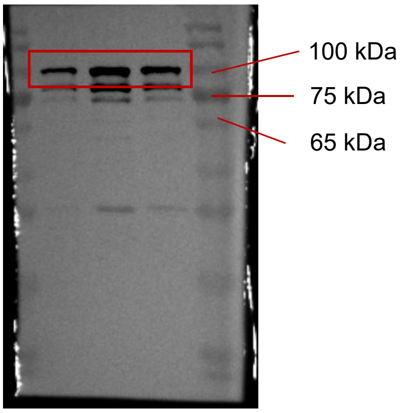

Trf 77 kDa

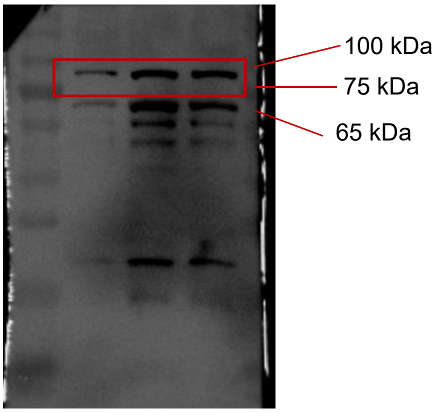

NCOA4 70 kDa

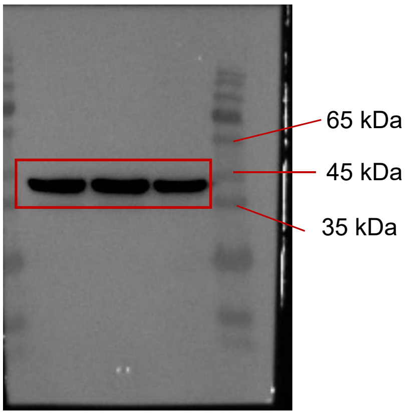

β-actin 42 kDa

kidney tssues   C、M、M+cur

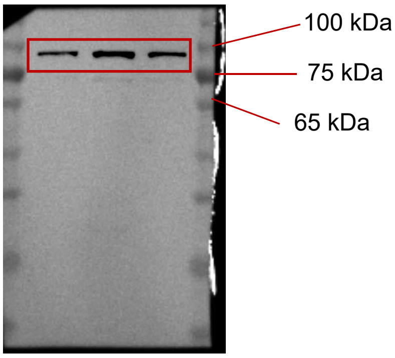

ACSL4 80 kDa

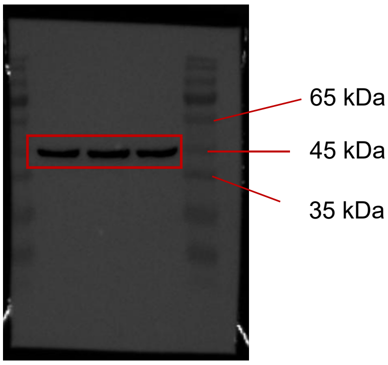

β-actin 42 kDa

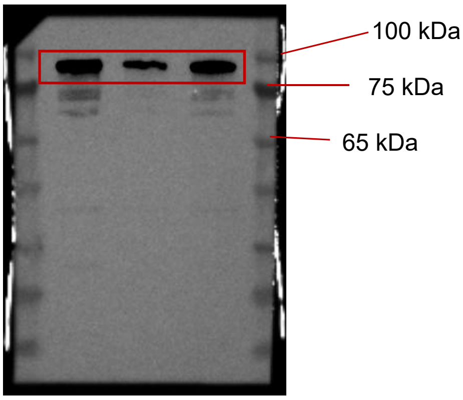

SLC3A2 80 kDa

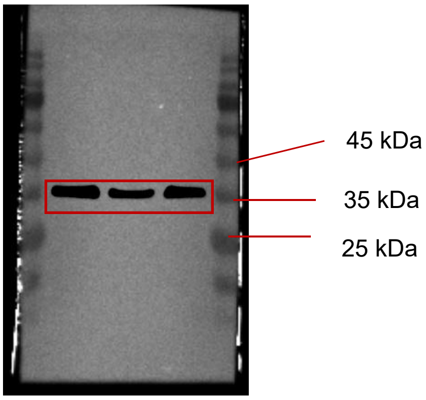

SLC7A11 35 kDa

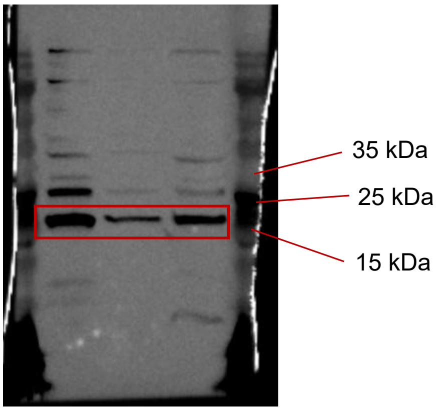

GPX4   21 kDa

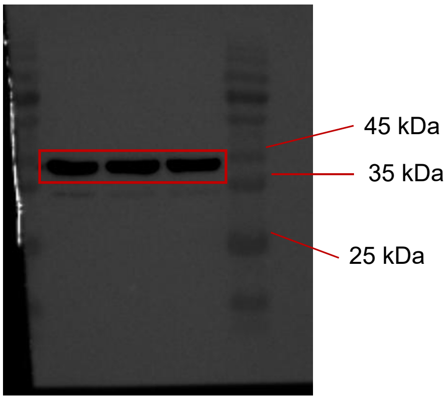

β-actin   42 kDa
